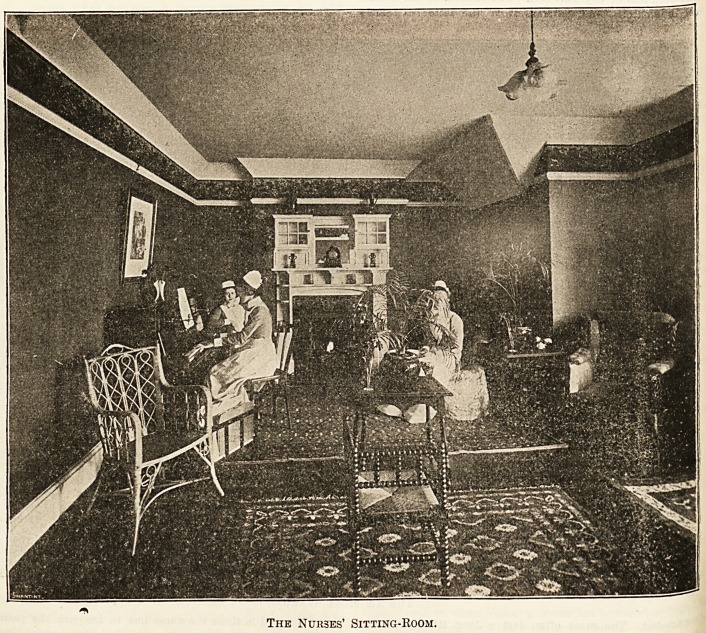# The Hospital. Nursing Section

**Published:** 1904-04-30

**Authors:** 


### The Hospital.

### Nursing Section.

Contributions for this Section of "THE HOSPITAL" should be addressed to the EDITOR, "THE HOSPITAL" NURSING SECTION, 28 & 29 Southampton Street, Strand, London, W.C.

No. 918.—VOL. XXXVI. *SATURDAY, APRIL* 30, 1904.

### Notes on News from the Nursing World.

### THE RECORD YEAR OF THE PENSION FUND.

THE increasing interest in the proceedings at the annual meeting of the Royal National Pension Fund for Nurses, which we report in full to-day, was shown by the presence of a large number of policy-holders. They had the satisfaction of hearing first-hand a story of continued and growing success. Not only were more policies issued by the Fund in 1903 than in any previous year since it was established, but, as the chairman pointed out, while the number of policies taken out increased, the individual amount assured was somewhat smaller, and the ages of the proposers were higher. The decrease in the withdrawals, the increase in the amount paid in pensions, the augmentation of the surrender value of a Pension Fund policy, the Fund's investments, its absolute safety, the certainty of the annuities, and other striking features, were clearly set forth, and we are sure that the thousands of policy-holders who could not be at the annual meeting will read the speeches with pleasure. Mr. George King, at the close of his illuminating remarks on the valuation, said that "it would be much to the advantage of the nurses if they would induce their friends to join the Fund and share in its success." This is very true. While the benefits which the individual nurse derives from joining the Fund are many and substantial, the existing members must necessarily gain by accessions to their ranks. In acting as missionaries of the cause they promote their own welfare, because it is identical with that of the Fund.

### NURSING TROPICAL DISEASES IN THE COLONIES.

AN account of the nursing of tropical diseases in London is given, with illustrations, in another page by the matron of the Branch Seamen's Hospital, from which it will be seen that there is now no reason whatever why nurses who desire to work in the tropics should not be thoroughly equipped for their duties before they leave these shores. At the admirable institution at the Royal Victoria and Albert Docks the three years' training, which can be started a little earlier than at some hospitals if a candidate seems capable, includes the nursing of many diseases which are common in the tropics—for example, beri-beri, blackwater fever, malaria, and that troublesome malady, guinea worm. The matron in her interview with Our Commissioner supplies some useful information, and points out the vital importance of nurses who contemplate proceeding to tropical countries being able to act for themselves, since they may at any time be in places where there is no medical man at hand. We are glad to hear that the Colonial Nursing Association frequently send their nurses for a three months' course to the hospital at the Docks. But the best plan for those who purpose to specially devote themselves to the nursing of tropical diseases beyond the seas is to become probationers at that institution. For while in the surgical ward the nursing is the same as that of a general hospital, that in the tropical ward is not only unique, but the experience gained during the time of training is the best possible qualification which they could possess.

### LADY LONDONDERRY AND DISTRICT NURSING.

AT the annual meeting of the Newtownards District Nursing Society, the Marchioness of Londonderry, who presided, introduced a proposition to broaden the basis of the Association. Having dwelt on the importance of adding to the staff a maternity nurse who could attend the mother for 12 or 14 days after the confinement, nurse her back to health, and give the little one a fair start in life, Lady Londonderry suggested that persons employed in the large works in the town should be asked to subscribe a small sum each. The suggestion, she mentioned, had been considered in several quarters, and favourable replies had been received. Subsequently, the Rev. W. L. Whetham, who expressed his gratitude to the President for having introduced into the working of the Society the principle of mutual help, moved the adoption of the proposal, and it was carried. The number of visits paid by the single nurse last year exceeded 4,000, and it is clear that she needs a colleague.

### COMPULSORY NURSES.

A PROPOSAL has been made by a lady doctor in Zurich that all unmarried girls belonging to the well-to-do classes should be compelled to devote a year to unpaid hospital or ambulance work. Of course, a year's training would not, as the author of the proposal seems to imagine, convert such persons into properly-trained nurses. But if it were suggested that the term should be sufficient to enable them to obtain adequate qualifications, our objection to the idea of compulsory nurses would remain the same. It is almost as bad as that of compelling young men who enter the Universities to take holy orders.

### MIDWIFERY IN FIJI.

THE chief medical officer of the colony of Fiji contributes this week the first of a short series of articles describing midwifery as it is practised in the islands where he resides. There is no higher authority on the question than Mr. Glanvill Corney, who has not only spent some years in Fiji, but was a member of the Commission appointed by the British Government to inquire into the causes of the decrease of the native population of the islands. One of these is their midwifery, of which Dr. Corney says its most redeeming feature is probably its prevailing policy of non-interference. It is quite a common thing for confinements to take place out of doors. The primitive conditions of child-birth in Fiji are in striking contrast to those under which it takes place in modern Europe, but Dr. Corney recognises compensations even in the loss of comfort.

### THE NURSES' CO-OPERATION.

The thirteenth annual report of the Nurses' Co-operation has been forwarded to us. At the end of 1903 there were 497 nurses on the general staff, and 20 asylum-trained nurses who take mental cases only. During the year 6,183 cases were attended, 1,391 being surgical, and 4,792 medical and mental. It is stated that the work in the lady superintendent's department "has been carried out most successfully and economically under the able direction of Miss Roberts." Since this statement was printed a new lady superintendent has been appointed. The income of the Society was £65 less than that of the preceding year, and it is explained by the addition to the list of nurses who pay only 5 per cent. on their earnings. The income derived from the commission on fees earned by nurses was £2,983. There is again a considerable loss on the Nurses' Home and Club. In any other business enterprise, those whose earnings keep the Society going would insist upon having an adequate voice in the management of its affairs.

### IMPORTANT CHANGES AT DERBYSHIRE ROYAL INFIRMARY.

It has been resolved to extend the period of training at the Derbyshire Royal Infirmary from three to four years. A portion of the fourth year will be spent, as in other large hospitals, in private nursing. In consequence of the growth of the massage, Greville, and Finsen Light departments, and also of the great increase in the number of in-patients, the nursing staff has for some time been considerably overtaxed, and the Governors have now accordingly decided to augment it by six. They have also determined to add twelve bedrooms and the necessary offices to the nurses' admirably-arranged home. The money for the purpose will not come out of income, but will be taken from legacies left to the infirmary.

### THE METHOD OF LIVING FROM HAND TO MOUTH.

In an appeal on behalf of the funds of the Aberdeen District Nursing Association, Professor Salmond, who presided at the annual meeting, said that it certainly should be no great strain upon the citizens to find the comparatively small sum which was necessary in order to make the accounts balance in a satisfactory way. No doubt this is true, but though there may be plenty of people in Aberdeen who could write a cheque for £144 without feeling the loss of the money, the difference between the income and expenditure of the Association is none the less a matter for regret. Another speaker at the meeting very wisely dwelt on the importance of avoiding the method of living from hand to mouth, which is too common with many charities. As the work of the district nurses, who last year paid 27,644 visits, is so generally admitted to be in all respects admirable, and is so much appreciated by the poor themselves that 101 patients contributed £33 10s. in the twelve months, there ought to be no difficulty in collecting a sufficient amount in regular subscriptions. If, as it was suggested, want of knowledge of the operations of the Association is the reason why adequate financial support has not been forthcoming, this should easily be remedied. The present supporters should be persistent advertisers of its merits, and care should be taken to bring its objects, the results of its labours, and its needs, systematically before every householder.

### STRUGGLE BETWEEN A NURSE AND HER PATIENT.

It is not often that a nurse has such a distressing experience as that of Miss M. G. Duffy, whose patient, a young lady of 21, last week deliberately committed suicide at Brighton. The deceased, it was stated at the inquest, had always been of an excitable disposition, and her father told the jury that the climax in her condition was reached owing to her disappointment at not hearing any news from a gentleman who had gone abroad and had promised to write. It was not until Friday morning that she became violent, and then she threw a basin of water over the nurse in the bedroom. Afterwards she became quiet, but suddenly got up and began to struggle with Miss Duffy. Being apparently the stronger, she forced the nurse out of the room, and, having locked the door, threw the window up and jumped out, falling on the bricks 36 feet below. She died almost immediately, and, the doctor having expressed his conviction that her mind had become unhinged, the jury returned a verdict of suicide during temporary insanity. A rider was added to the verdict that no blame attached to anyone.

### ASYLUM WORKERS' ASSOCIATION.

The annual meeting of the Asylum Workers' Association will be held on Tuesday, May 17, at 11 Chandos Street, Cavendish Square. Sir James Crichton Browne will preside. It is announced that the gold medal for 1904 for long and meritorious service has been awarded by the Executive Committee of the Association to Mr. W. Headon, Devon County Asylum. The three silver medals have been awarded to Mr. T. Alexander, Notts City Asylum, Miss E. Atkins, Caterham Asylum, and Miss E. Gribble, Holloway Sanatorium.

### AN EXCELLENT COMMENCEMENT AT BATLEY.

The result of an effort to form what is called the Batley Ambulance and Nursing Service is that upwards of £100 has been subscribed. A representative committee has been formed, and it has been determined to secure the services of a nurse who has had three years' hospital training and three years' experience in district nursing. All the nursing is to be done free of charge, if, as there is every reason to anticipate, the Batley people will provide a sufficient income for the purpose. It is hoped that by enlisting the interest of the local friendly societies between £130 and £140 will be got together before the project is financially launched. We notice, with satisfaction, that several of the larger subscribers have promised to subscribe for three years.

### LIVING ON CAPITAL.

At the annual meeting of the Cornwall County Nursing Association, the chairman stated that the organisation were living on their capital, and the figures of the report show that the expenditure during last year was £660, as against an increase of £490. The chairman stated that he was told that the best thing the association could do was to spend all the money they had, and then, when they had got to the end of the tether, have a bazaar. But supposing that the bazaar failed to yield anything like the sum required? In any case, the idea of depending for income upon such a source as this is as risky as it is improvident. If the work of the association is so much appreciated in the county that, as one of the speakers said, the removal of any of the nurses has become impossible, there should be no serious difficulty in augmenting the regular income. It has been decided to raise the affiliation fee from 5s. to a guinea per annum, but this will only make a small difference. If the Cornish people think that the County Association is indispensable as well as the district associations, they should be pressed to find the requisite money to keep it solvent.

### LONDON SCHOOL NURSES.

The President and Vice-Presidents of the London School Nurses' Society are again making an appeal to the public for more money to carry on their work. Unfortunately, the recent educational changes in London have had a disastrous effect upon their funds. Managers, who formerly subscribed for a nurse in their schools, have shown themselves inclined to withhold their subscriptions until more secure of their own position under the new authority. It is also uncertain whether the three nurses who were appointed "experimentally, and for one year," by the London School Board last November will be continued by the present management. The result of this uncertainty has naturally been to greatly discourage subscriptions. And yet the need of the children is just as great, and it would be a matter for much regret if the one half-time and the five full-time nurses in the employ of the Society who at present visit 80 schools, should have to be reduced in number, or run the risk of being dispensed with altogether.

### INCREASE OF SALARIES AT DARLINGTON.

The Darlington Guardians have wisely decided to increase the salaries of their nurses. At the last meeting of the board the workhouse committee reported that they had selected five nurses from the list of applicants for the vacant situations at the workhouse to appear before the board, but that three had since written stating that they would not come. Moreover, none of the applicants appeared at the meeting, and the clerk also reported the resignation of another nurse. In these circumstances, Major Priestman expressed his opinion that the salary of £25 a year offered was too little, and it was ultimately determined to advertise for three nurses at a commencing salary of £30, rising to a maximum of £35. This may not be all that is necessary, but it is a step in advance, and as the question of better apartments—which are badly needed—has been referred to the workhouse committee, it may be hoped that in a short time the Darlington Guardians will likewise be able to provide more comfortable accommodation than is at present available. The town of Darlington is so thoroughly up to date, and the surroundings are so pleasant, that, other things being equal, there is no reason why nurses should be at all inclined to give it the cold shoulder.

### FRIENDLY SOCIETIES AND DISTRICT NURSING.

A pleasing feature in connection with the annual report of Hanley Nursing Society is that last year the Amalgamated Friendly Societies increased their subscription. Mr. Newton, speaking on behalf of the societies at the meeting, said that the work of the nurses had their heartiest sympathy. From first giving £10 10s., the societies had increased the amount to £15 15s., and they would do their best to keep it up. "The nurses," he continued, "earned very great praise from those whom they visited; they performed their duties splendidly, and were doing a very grand and noble work in the town." We are glad to see, moreover, that the balance in the hands of the treasurer of the Nursing Association at the end of 1903 was £28. An addition to the staff appears to be required, and, judging by the enthusiasm which prevailed at the meeting, there will not be very much difficulty in raising the extra income needed to defray her salary.

### AMALGAMATION OF ORGANISATIONS AT HEREFORD.

At the annual meeting of the Hereford Nursing Association it was proposed by the Bishop of Hereford that the committees of the Association and the Maternity Society should appoint a joint committee to take into consideration the question of amalgamation. Several speeches were delivered by members of both organisations, and no opposition was offered. But one official of the Maternity Society suggested that some of the subscriptions might be lost by amalgamation. This is quite conceivable, and in the event of the amalgamation being determined upon, it will be very necessary for the authorities to make it clear to all subscribers individually that the reduction in expenditure will be trifling, but that the two societies working as one may be able to render more efficient service. The experiment has already been made at Gloucester, and the results have been, not only an increase in the sphere of usefulness, but also an addition to the funds.

### A DEFICIENCY AT KETTERING.

Notwithstanding that the work of the Queen's nurses in Kettering continues to increase, 391 cases having been attended and 12,057 visits paid last year, the speakers at the annual meeting of the Kettering District Nursing Association have to lament that the receipts were not sufficient to cover the expenditure. The deficiency is £41, and though the Association has still a balance in hand at the bank of £75, this will, obviously, soon be swept away if special efforts are not made in the town to increase the subscriptions. It appears from the remarks of the treasurer that it is not fully understood that, while the Queen's nurses cannot accept any gratuities in cases which they attend, the Association is always glad to receive contributions from people in Kettering who have benefited by the visits of the nurses. We may go a little further and affirm that persons who are nursed, or whose friends are nursed, under the auspices of the Association, ought to make it a point of duty to subscribe something, however small, in order to maintain the organisation which enables them to enjoy the advantages of skilled nursing.

### The Nursing Outlook.

"From magnanimity, all fear above;
From nobler recompense, above applause,
Which owes to man's short outlook all its charm."

### MALE NURSES AND REGISTRATION.

The calm indifference of the male nurse towards the two Registration Bills now before Parliament can only be due to the knowledge that the good worker is always sure of his hire, and that you cannot make good workers by Act of Parliament. For though there are not many male nurses in general work, there are great numbers engaged as mental attendants, and there is no doubt that if either of the Bills passed as they are now, the mental nurse would be obliged to come under it, or else to drop the word "nurse" and take only the title of "attendant." Even then the legal position would be extremely difficult, and it would be necessary to define, for instance, in the State Registration Bill, the meaning of the phrase "nursing of the sick."

There is one thing certain, namely, that the avocation of nursing will always be chiefly in the hands of women, but the male nurse will always be a factor worthy of professional consideration. Doctors sometimes prefer male nurses for male patients, and since the South African war there have been many more male nurses procurable, who with a wide experience of nursing, have also some experience of "making things do." In one case which lately came before our notice there were two women nurses in constant attendance for three months and then, at the earnest request of the patient—a young man—an old soldier of experience was procured in their stead. The results were fourfold: (1) Ease of mind to patient in being attended by one of his own sex. (2) Brightness of mind to patient who found conversation about war and sport more to his taste than gossip about "matron" and former patients. (3) Expenses were reduced by two-thirds; not only was this because the one man did the work of the two women easily, but the chemist's bill and all other expenses were reduced; there was economy instead of extravagance. (4) Harmony in the domestic arrangements—the male nurse never had any friction either with the servants or the family. The success of the male nurse at present is largely due to the fact that he only takes up the work from inclination, he is generally looked down upon and therefore does not suffer from self-esteem; he has not been trained into a machine.

But after the two years of grace in the event of the passing of either Bill, the male nurse will have to produce evidence of three years' training, and that will be very hard to do. For the arrangements for the training of male nurses are very meagre at present: with the exception of the military hospitals, there is only the National Hospital for the Paralysed in England which deliberately sets out to train male nurses; and in the United States there are only the Bellevue Hospital, the New York City and the Grace Hospital at Detroit. So far there has been no unfriendly rivalry between men and women nurses—because, as a physician suggests—the women have it all their own way! But obviously it would be unfair to ask men to come under the suggested legislation without providing the necessary means of training; and also it would be unfair to women to ask them to go through all these necessary qualifications and expenses before allowing them to call themselves nurses, and to leave the men out of the Act altogether and let them call themselves nurses and act as nurses, whether qualified, or trained, or not. Under the second state of affairs the men would probably largely oust the women.

The situation is worth considering, and yet the Association of Asylum Workers has in vain asked men mental nurses to consider it—they are not in the least interested. They evidently know there is always a loop-hole of escape, and that the day of registration is yet far off. One laughable example of the loopholes of escape comes to us from Cape Town, where an English matron was amazed at the ignorance and general unsuitability of a "registered" sister she found at work in the wards; on inquiry the matron discovered that this nurse had gathered her three years' experience in five different hospitals, breaking her contracts just when she felt disposed, and had then qualified by passing the Cape Government Examination, and had applied for and been appointed a hospital sister.

On June 27th last, we dealt in these pages with some of the difficulties of legislation, and the *Medical Record*, of New York, had a leader pointing out that our sharp criticism of the United States Nurses' Act was deserved. Our contemporary said—"In no State but New York has an Act been passed likely in any way to improve the status of nurses, and the New York Act is by no means an ideal one;" and continued—"The status of the nurse at the present time is ill-defined, and the whole question of nursing is in an unsatisfactory condition." If this is the editorial comment on the American Acts, and if the result of the Cape Town Act is the production of nurses of the stamp quoted, we are not astonished at the apathy of male nurses in England. But it is not wise to let apathy go too far; the better organisation of nurses can certainly be furthered by discussions on the present and possible future state of affairs. Male nurses and mental nurses ought to think out the subject, and decide whether they want legislation; and if they do want it, in what form it ought to come.

### The Modern Nursing of Consumption.

By Dr. Jane H. Walker, Physician to the New Hospital for Women, London, and Medical Superintendant of the East Anglian Sanatorium, Nayland, Suffolk.

### LECTURE IV.

### Nursing in Sanatoria.

In a sanatorium, of course, the nurse has fewer responsibilities than when she is nursing in a private house. The patient's daily life and the nurse's daily duties are mapped out by the doctor, who is always at hand. A nurse's work may be thought to be passed in carrying out rather trifling details, but it possesses the advantage of not being specially arduous, and therefore it can be undertaken by women who are too delicate to go through the regular hospital course.

This brings us to the subject of training. We would not say, as one doctor at a sanatorium has said, "I want automata, not nurses," and who therefore employs people of the regular servant class. At the same time the ordinary hospital-trained nurse is not of much use in a sanatorium for better class patients. She is too apt to be full of preconceived ideas, and does not realise that a very large part of the cure depends upon cheerful surroundings. But some training is certainly necessary. It makes a great deal of difference to a patient to have his bed made properly, to be skilfully washed in bed by experienced hands, and to be otherwise attended to by some one to whom the whole thing is a matter of course. These things, which are factors common to all nursing, can, we think, be learnt in six months' training in an ordinary hospital. Such is the plan adopted in the nursing department of the sanatorium with which we are connected. A very useful thing, which is being gradually evolved at the present time, is a training school for nursing on the rational method for the treatment of consumption. Not infrequently, for one reason or another, it is necessary for a consumptive patient to remain at home, and for such it is advisable to provide a so-called "open-air nurse." Of course, in choosing nurses for consumptives one should remember that they should be of specially cheerful dispositions, should be possessed of considerable tact, and should recognise the fact that consumption is not an incurable disease.

### The Duties of the Nurse.

What are the duties of a nurse at a sanatorium? Alas, to a nurse whose idea of work is the accident ward of a London hospital, they seem tame. A few years ago there was a craze for going to Norway to learn the Sloyd system of wood-carving; a friend who was bitten with this idea was one of the earliest pupils at Naes, and on her return, when asked to describe the system, said, "Oh, it is mainly whittling." That rather describes a nurse's duties at a sanatorium. Little that is exciting, not often a sudden and great rush of work, often possibly no real nursing as an ordinary hospital nurse understands it, and yet it is a nurse's work, and not a housemaid's. A new nurse lately said to the matron of a sanatorium, "But this is only housemaid's work. I am passionately fond of nursing, but this is not nursing." This nurse later on, when discussing her work, acknowledged that she saw, as she expressed it, that "even dusting can be done so as to make it desperately interesting."

### A Day in a Sanatorium from a Nurse's Point of View.

A nurse gets up at about seven, has her breakfast as soon as she is dressed, and then is ready to take breakfasts at eight to all the patients who are in bed. The question of food at sanatoria for consumptives is an all-important one, and cannot be too strongly insisted on. The patient has been seen by the doctor before breakfast, and any special diets or amounts of food regulated. Whatever has been decided on must be faithfully carried out, and it is an absolute rule that no plate must be taken away from a patient till all food on it has been eaten. The nurse's business is to see that this rule is enforced. If for some reason the patient cannot finish his meal, then the matter must be referred to the doctor. This rule applies equally of course to dinner and supper.

*After Breakfast.*—When the breakfasts have been given, the rooms of those patients who are up and at breakfast should be seen to. If it be winter and the windows have been shut for the patients to dress, they must of course be opened at once, and if the radiators have been on, they should be turned off. The bed must be stripped and the slops emptied by the housemaids, who should also take up the strip of carpet by the bed and put it outside the room, and the floor must be swabbed with water every morning. The nurse must see that the housemaids carry out these duties properly. The beds should be left unmade as long as possible; and when they are made, patients' idiosyncrasies in the matter of the disposal of their bed clothes and pillows should be attended to and marked.

*The Importance of a Wet Duster.*—When the floor is swabbed and the bed made then the nurse can do the dusting. It is done with a wet duster. We say wet, advisedly, experience having taught that a damp duster is apt to become dry and so to defeat its own ends. The object of using a thoroughly wet duster is twofold Firstly it does its work more efficiently, and secondly it prevents any dust from flying about the room or falling on the floor or bed. Although, under the careful conditions in which patients are kept in sanatoria the dust as a rule probably contains no tubercle bacilli yet, supposing that any should have been carried about the room by the air, it is well known that they can live for a long time in dust, and when dry can be carried about by currents of air from place to place. When a wet duster is used, not only are the bacilli more readily taken up, but being damp they cannot be blown about. Also dust, *per se*, is injurious to phthisical patients and therefore the removal of all dust particles must be as thorough as possible.

*Rest and Dinner.*—When the patients in bed have finished breakfast they will need either to be washed entirely or to be assisted in washing themselves. In all these particulars of course the doctor's orders will be followed. Some patients, who are mostly in bed, will be allowed up for a few hours daily, lying on the long couch which is an integral part of the furniture of every room. The patients will require to be settled comfortably, with books, papers, and any other requirements by the side of the bed or couch.

Every room must be made clean and tidy by 12 o'clock, as at that time each patient goes to his room to rest for an hour before dinner. During this hour the patients are visited by the doctor, and the nurses have their own dinners. It is a good plan for the matron or head nurse, if there are a large number of patients in bed, to carve and send up the meals half an hour before the regular meal in the dining-room, but otherwise the food is served out by the doctor from the dining-room and taken by the nurses to all patients in bed.

After dinner, which takes from one and a-half to two hours, the trays are fetched away, and as many nurses as possible go off duty. Those who are on usually have a slack time till they have to prepare the patients in bed for their suppers and get their trays ready.

### THE MODERN NURSING OF CONSUMPTION—*Continued.*

From six to seven P.M. is again rest time. From seven till bed-time every nurse is generally fully occupied. Many patients have to be sponged all over before settling to rest, some require help with their baths, and all patients in bed need assistance in washing and preparing themselves for the night.

*Feeding the Patients.*—An important part of a nurse's duties frequently is feeding the patients. In any severe cases of phthisis, it is important to reduce all fatigue as much as possible, and patients must therefore be spared any unnecessary exertion. By feeding is meant actually putting the food into the patient's mouth. To some patients this is a vexation and annoyance, and requires to be done with the utmost care and tact. To many it is the greatest relief and boon. Some patients are helped by the presence of a nurse while they are eating, or if the nurse read aloud an amusing book, it will often make the patient take his food with less pain and toil.

All these duties performed, the nurses are finally off-duty between half-past nine and ten o'clock. The whole place is quiet, and everyone has retired to rest by ten.

*No Night Duty.*—In a sanatorium there is usually no need for any night nurse. Nurses must have their bedrooms in such a position that they are in easy communication with the patients under their charge and all patients' bells must ring into the nurses' bedrooms. As a matter of fact, in most sanatoria nurses are rarely rung up at night, and of course if an exceedingly bad case be under treatment, special arrangements will have to be made for night nursing.

With regard to the times off duty, every one must be off duty for a short time every day. But it is more important to give every nurse a chance of having twenty-four hours off duty once a month. The situation of a sanatorium is generally isolated, and the work apt to be monotonous, therefore a sufficient time to enable a nurse to get into entirely different surroundings is advisable.

In staffing a paying sanatorium with nurses, one nurse to every six or seven patients should be allowed for and if many patients are entirely in bed, occasional extra help will probably be required. The total staff required is about one to every two patients.

### Encouragement to Beginners.

LOOKING through my old note-book, I have found the notes of my first lecture, which I attended as a shivering little pro. I can smile at it now; but oh! what I suffered that night as I followed the other probationers into the lecture-room! I was not reassured by the usual diagrams of the skeleton, hanging round the room, the specimens of bones laid out to view, and the vivid pictures of the internal arrangements; they nearly made me sick. I put on as bold a face as possible, trying to copy the unconcerned looks of the others; but when the door opened, and we rose at the entrance of the doctor and matron, I would have given a year of my life to run away, but escape was impossible, as, being the junior, I found myself seated near the matron. Having taken the advice of a pro. six weeks my senior, I had provided myself with a huge manuscript-book and a sharp pencil, but my sharp pencil was not inspired, judging by the look of the notes now. No doubt the lecturer was equal to the occasion, and gave a graphic and clear explanation of his subject. I wrote for my life, feeling in a hopeless muddle, but fully expecting that my notes when read over at my leisure, would be found all that was necessary. To encourage pros. in a similar position, and to make many difficult problems easy to them by my lucid explanation, I will pocket my pride. Here is

### A COPY OF THE NOTES.

"Compound fracture similar to simple. In former times the air entered a wound. May cause putrefaction, lead to Pyemia, but owing now to antiseptic dress not so long healing owing to the exclusion of air.

"Callus bony ring around nature absorb its bony matter You have ——. In a broken bone set so as to prevent motion.

"Muscles are possessed of great power. Symphol of frœdic (probably symptoms of fracture) grating sound called crepitus—two ends of bone cold grate in fracture. Secure your fracture as quickly as possible. Umbrellas, sticks, or anything, thick rolls of paper.

"Bones are divided into short areas, flat bones, bones of the skull, bony matter. If you have a wound in connection with the joint you will have a greasy feeling which will never ——.

"Joint oil prevents inflammation. Beginning of bone—formed of bone called cartilage—first begun of the bony matter, it increases until it occupies the whole of the shaft. All bony matter become growth of bone takes place.

"You may, if not careful, break off separation of the spine. May be separet like a fracture. Separation of ephisis in the 20th year it becomes quite joined. The earthy matter and animal matter equal, in young more earthy matter becomes deposited.

"Fractures may be divided into simple compound complicated with other impi.

"A simple fracture may be obliging, it may split lengthway.

"Green stick fracture.

"Complicated may enter the lung."

### THE SECOND EFFORT.

This must not be taken as an instance of my powers of spelling, as I really could spell before I began to take notes. It is the breathless haste that does the mischief and takes away the little sense one ever had. Being determined to master my subject before the next lecture, I bought myself a "Blackie's Physiology," and in my off-duty time tried to learn the first chapters by heart, even carrying the book into the chapel, and sleeping with it under my pillow, hoping to wake up extra early, which I never did, needless to say. Having so well primed myself, I had not so many fears at the next lecture, for what could they tell me more than I knew? The matron was the lecturer this time, and she proved more terrifying than the doctor, for she actually asked questions. Fancy my feelings when she started with, fixing her eye upon me—"Nurse M——, how many bones are there in the human frame?" I grew very hot and stammered out, "I don't know, Matron." She, in the kindness of her heart, wishing to give me a chance, continued to ask me the easiest questions, to all of which I could only give the same answer, from sheer fright. At last I did manage to tell her, to her pleasure and my relief, that arteries were round and spurted when cut, and that veins oozed and were flat! I remember I was congratulated afterwards, and clapped on the back at this wonderful exhibition of knowledge. Long afterwards I really was surprised to hear that our kind matron, who so frightened me that night, had said of me that I "was as sharp as a needle"—but then she always was so encouraging to the beginners.

### Nursing Tropical Diseases in London.

INTERVIEW WITH THE MATRON OF THE SEAMEN'S HOSPITAL AT THE VICTORIA AND ALBERT DOCKS.
BY OUR COMMISSIONER.

The latest development at the Seamen's Hospital at the Royal Victoria and Albert Docks is the opening of an isolation building. This on the occasion of my visit to the valuable branch of the Dreadnought which faces Connaught Road Station in the heart of the dock district, had only just been completed, and when I inspected it under the auspices of the Matron, Miss Graham Knight, it was absolutely spick and span. The importance of the addition, which consists of a small ward for two, a nurse's room, a kitchen, and every other convenience, may be imagined from the fact that some time ago, when a case of plague occurred in the hospital, the greatest difficulty was occasioned in isolating the patient. Even since the large new wards were opened, the single-bedded wards in the main building have had to be utilised for cases of infectious disease.

"We have realised the need of proper isolation quarters for some time," said the Matron, as I admired the absolute completeness of all the arrangements, only expressing regret that a large flat roof had no been made accessible from the

The Seamen's Hospital at the Docks.

nurses' room, "and, of course, it might become urgent at any moment."

"What infectious maladies are most dreaded?"

"Plague, cholera, and yellow fever, which could not be removed."

Then we passed into the most interesting ward containing the sufferers from tropical diseases, and I was able to obtain an idea of the nature of the experience gained by the probationers at the hospital. Both here, and throughout the building, there is every modern appliance; and before we finished our task of inspection, including a peep into the headquarters of the London School of Tropical Medicine, I went into the modern mortuary, with its separate viewing-room, which was added quite recently.

### The School and the Nursing.

"The great development of the hospital took place when the London School of Tropical Medicine came here?" I observed to the matron.

"Yes; the two large wards were added then. The hospital itself was opened in 1890, but at first there were only 23 beds. Now there are 50 beds, distributed in two wards of 18, two of six, and two private rooms. The opening of the school has had a great effect upon the hospital."

"And also, I gather, upon the work of the nurses?"

"Unquestionably. At the outset the probationers were only trained for two years, but in 1901 we extended the period to three years."

"That was after you became matron?"

"A couple of years after. I came five years ago from the Seamen's Hospital, at Greenwich, where I had been surgical sister. I was trained at the London Hospital."

### The Nursing Staff.

"Has the nursing staff here always been distinct from that at Greenwich?"

"Yes, entirely. At the present time our staff consists of two sisters, six staff nurses, and eight probationers. The staff nurses are in their third year of training, and the senior is in charge of the extensive out-patient department."

"The sisters, of course, were trained elsewhere."

"The day sister was trained at the London Hospital, and the night sister at Croydon Infirmary under Miss Julian."

"Do your probationers come for a month on trial?"

"For two months. At the end of that time, if suitable, they enter for three years, receiving salary and indoor uniform. The salary is £12 the first year, £15 the second, and £20 the third."

"And the age of admission?"

"It is supposed to be from 23 to 30. But a hard and fast line is not drawn. I do not object to take a girl who is a little younger than 23 if she seems capable."

"How far does the theoretical training differ from that of an ordinary general hospital?"

"Special lectures are given on tropical work. As to the rest the members of the medical staff deliver lectures on the usual subjects and an examination is held at the end of each course of three months."

### The Special Work.

"I should like you to tell me as much as you can about the work in the tropical ward."

"That, as you know, is the feature of the nursing at this hospital. One of the most serious diseases we nurse is sprue, and in these cases a nurse has to exercise both the utmost patience and the utmost vigilance. Owing to the nature of the disease the patients are inclined to be very irritable and unreasonable, and special care is necessary to keep them from taking a chill, and the temperature of the ward must never be under 70 degrees. Then there is the feeding, as it often largely depends upon the feeding whether the patient will recover.

### Nursing Beri-beri.

"You have some very bad cases of beri-beri?"

"Yes, frequently. We get an increasing number of cases. The chief danger in beri-beri is the heart trouble. The patients have, therefore, to be kept very quiet in bed. When some of the patients are brought in they do not look at all ill. Notwithstanding this, we have had them die in an hour. Therefore a nurse must perpetually watch the symptoms. If the heart trouble is very bad the nurses are instructed to give inhalations of nitrate of amyl until the doctor arrives. As to feeding, it is impossible to be too careful in respect to the restrictional fluid diet, which is a pint in 24 hours. Nothing bulky, such as rice, must be given."

"It is not an objectionable disease to nurse?"

"Not in the least.

"Is there any particular nationality prone to the complaint?"

"Many of the patients are natives of Zanzibar, and the disease is chiefly confined to sailors. We have cases of wet beri-beri, and of dry beri-beri. The victims to the former are œdamatous, and sufferers from the latter extremely emaciated. The cases often last a long time, perhaps as long as two months.

"Are all the patients here adults?"

"Yes, men of varying ages. We have a great number of cases of dysentery, some very acute. In these cases attention in the matter of diet is also essential. The patients are put on milk and barley water, and no solid food is allowed. Deaths from dysentery occur very rarely here."

Malta and Blackwater Fevers.

"Do you have many cases of Malta or Mediterranean fever?"

"A considerable number, and the disease is rather trying, because a relapse so often takes place. Thus, the high temperature may come down for a week and then go up again; and these alternatives may last for three months. The nurse has to sponge the patient frequently, and if the pains are very bad, she administers fomentations in order to soothe them. Milk diet is given until eggs or chicken broth can be taken. There is just now a Norwegian sailor suffering from this fever, and we lately had a naval lieutenant down with it. Then there is blackwater fever."

"Are there any special precautions necessary in nursing cases of blackwater fever?"

"In severe cases, which are serious, there are three things to look out for, namely, syncope, hyper-pyrexia, and suppression of urine. These are very anxious cases and require most careful nursing.

Malaria and Guinea Worm.

"Of course you have malaria cases?"

"Yes, and in these the nurse has to ice-pack the patient if he suffers from hyper-pyrexia. In other cases, simple sponging is employed. The nurses are taught to administer intra-muscular injections of quinine so that they may be able, if they go to the tropics, to know what to do in an emergency."

"What other diseases are nursed in the tropical ward?"

"Guinea worm is one. Perchloride of mercury is injected into the body of the worm which kills the parasite and in a short time extraction is generally effected. The disease itself is common in different parts of the tropics, but our patients are chiefly natives of Bombay. We have also a large number of cases of liver-abscess, which are usually operated upon.

The Nationalities of Patients.

"I suppose that the accident and surgical ward is generally full?"

The Tropical Ward.

"Yes; and we have many very severe cases. The majority of the patients are British dock labourers, but we get a few Norwegians and Swedes."

"You have, I see, various nationalities represented in the tropical ward; but I did not notice any Chinese patients."

"No; but we are very seldom without Chinese. They are usually suffering from beri-beri. We have Japanese, Zanzibarese, French, German, Danes, Norwegians, Swedes, Russian Finns, and natives from Bombay, Calcutta, and other parts of India. The darkies are most grateful patients; we treat them almost like children. They behave very well in the wards, and are not at all quarrelsome."

"No, it is just the same as that in the surgical ward of a general hospital. But it is a great advantage to the probationers that they combine the training of a general hospital with training in the nursing of tropical diseases which is unique in London."

"Are their hours similar to those in general hospitals?"

"The day nurses are on duty in the wards at 7 A.M. and off at 8 P.M. They have two hours off duty during the day and are allowed half an hour for lunch, dinner, and tea. The probationers get a day off once a month, and four hours on Sunday; the staff nurses half a day weekly, four hours every Sunday, and a Sunday once a month; the sisters two hours daily, half a day weekly, and Saturday to Monday once a month."

The Nurses' Sitting-Room.

"Can they make themselves understood to the nurses?"

"They know a little English, and the nurses know a few words of Hindustanee. But the darkies cannot always talk to each other owing to the fact that they speak different languages."

"What becomes of them when they are discharged?"

"The interpreter at the docks brings both the natives and Chinese patients here, and when they are ready to be discharged, we send for him, and he takes them off to their ships again. The Japanese, whom we find singularly intelligent, do not need looking after in this manner."

Hours of Duty.

"There is nothing unusual in the nursing in the surgical ward?"

"What are the arrangements for night duty?"

"The night sister has the assistance of two staff nurses and one probationer. She herself gets one night off during the month, and I hope to manage her a second. The night nurses are on duty from 8 P.M. to 8.30 A.M. They have two meals during the night, dine at 9 A.M., are off duty from 9.45 to 12.30, go to bed at one, and breakfast at 7.40 P.M. The probationers have three weeks' holiday, and the sisters a month.'

The Nurses' Quarters.

"Do you consider that the work is heavy?"

"We have spells of heavy work, but the strain is not so great as it is in a larger hospital. As to recreation, the nurses have to find it chiefly for themselves, though we have an asphalt tennis court. Theatre passes are granted pretty often."

"The nurses' quarters seem to be both comfortable and convenient."

"Yes; I do not think that there is any cause for complaint. Meals are all served in the dining-room in the hospital, and they have a large sitting-room of their own. Nearly all of them have a separate bedroom of a good size, and the whole of the rooms are nicely furnished. The entire building is lighted by the electric light."

"Is the little chapel used for the nurses exclusively?"

"Yes. The chaplain attached to the hospital holds services in the wards, and comes every day to see the patients. The Danes, Swedes, and Norwegians have their own missionary."

"Has the establishment of the school affected the number of applications for probationership?"

"We have plenty of suitable applications, and there is no doubt that the existence of the school, and clinics in the wards, is a great advantage to the nurses. I may mention that the Colonial Nursing Association send their nurses from time to time for a three months' course; they attend the lectures and do practical work in the wards. One of them, who has been in Sierra Leone, came for a second course."

Nursing in the Tropics.

"When your probationers finish their course is it probable that they will continue their career in the tropics?"

"That is the idea, generally speaking. But I consider it is essential that as soon as they get their certificate here they should go in for midwifery training. Then they ought to be able to get on well in the tropics or elsewhere. It is of vital importance that they should be able, if they contemplate proceeding to tropical countries, to act for themselves, since they may at any time be in places where there is no medical man at hand; and the object of qualifying them to rise to the occasion, in case of emergencies, is steadily kept in view in the training here."

"Finally, what in your judgment are the special qualities indispensable in the nursing of tropical diseases?"

"Much the same as in other nursing, but especially keen observation is necessary, and a nurse who is naturally observant, even of the smallest details, starts with at least one thing decidedly in her favour."

### A Funny Experience in District Nursing.

Some time ago, when I was district nursing in a small country town, I had a funny experience. Shortly after I settled in my new district I received an application from a respectable-looking woman, who asked me to go and see her father—an old man of 82—whose legs had taken "bad ways." She told me the old man's history, which was anything but creditable. He was, and had been in lodgings for 13 years. His wife was dead, and none of his family could put up with him. His daughter wished him to go into the infirmary but dared not suggest it.

I went to see the old man and found him sitting in front of the fire, drinking a little whisky. I tried to lead him into conversation. He was anything but communicative, but after some time he told me of his poor legs. They had been burned two weeks before, had had no attendance, and were sloughing badly. As he wasn't in a fit state to be up, I persuaded him to go to bed and let me examine his legs properly. I had to go out of the room while he got into bed. I wanted to help him to undress, but he strongly objected. However, after a little coaxing, I managed to wash him and ease his pain a little.

I found that the poor patient could not possibly be nursed in his lodgings, and tried to persuade him to go into the infirmary. He objected at first, then promised to go if I would go with him. I consented to take him in the morning if the parish doctor gave permission. I left a message for the doctor, and he readily agreed to his removal.

Shortly after I left the house, the Rector called to see the poor old man, who was beaming with satisfaction, and almost cried out, "Oh, sir, you be kind sending your good lady of the church to see me. Her ain't a bit like we poor mortals, her's so clever and looks so fresh and clean."

Our association was undenominational, and the clergyman did not know "the lady of the church" until the patient explained that the lady wore "passon's dress."

As soon as my evening round was done, I got the order from the doctor and saw the relieving officer, who ordered a cab to call for me at 8.45 the following morning, and drive me to the patient's lodgings. I waited for nearly half an hour after the time. No cab appeared. I then went to look for it, and was met by the wedding cab in full decoration. I asked for an explanation, and was told that I had to wait till the weddings were over, and there was no other cab available that morning. So in I got, grumbling at the delay, gave the driver the address, and we set off. We had scarcely gone 50 yards when a band of children ran after us, shouting, "A wedding! Hurrah, hurrah! Nurse is going to be married." As the horse ran swifter than the children, we were able to leave them behind before we arrived at our destination.

The patient was dressed, and waiting for me. He was surrounded by neighbours. I asked one of them what the doctor had said about him, and she informed me that the doctor said that old Tom's legs were "'illustrated,' and he'd have to keep 'em up."

With some difficulty we got the old man into the bridal chariot, and I asked the driver to go slowly, as the patient was rather exhausted, and I tried to steady the patient while we were going over the rutty ground. While so engaged another shout of "A wedding!" got up, and a rush was made for a sight of the bridal pair. As the windows were open there was no difficulty of getting a look. The poor patient, in dirty old clothes, and I in uniform formed a contrast as great as the difference in our ages.

The expressions on the onlookers' faces were an interesting study. Some of them followed us, and saw the cab turn in through the Union gate, which must have puzzled them somewhat.

I handed the order for admission to the porter, who gruffly inquired: "What relation is he to you?"

I was too amused to answer, but the patient answered, somewhat proudly, I imagined, "Her ain't related to me, I'm one of the Smiths of New Road."

Two porters carried my patient off, and then I sank back in the cab, overcome with laughter, wondering what next would happen to upset my mental equilibrium.

### To Nurses.

We invite contributions from any of our readers, and shall be glad to pay for "Notes on News from the Nursing World," or for articles describing nursing experiences at home or abroad dealing with any nursing question from an original point of view according to length. The minimum payment is 5s. Contributions on topical subjects are specially welcome. Notices of appointments, letters, entertainments, presentations, and deaths are not paid for, but we are always glad to receive them. All rejected manuscripts are returned in due course, and all payments for manuscripts used are made as early as possible after the beginning of each quarter.

### Beyond the Seas: Nursing at the Anglican Mission Station, River Mambare, British New Guinea.

This mission is 12 years old, and at first there were only the priest and laymen to attend to the sick in the villages, because it was nearly five years before a nurse came into the mission; since then she has been kept busy in the dispensary attached to the chief station (Dogura), where village natives are treated as out-patients. To begin with there was a great deal of malarial fever amongst the staff, a very small one then; and when the villages were visited with such epidemics as influenza, whooping cough, etc., everybody was overworked. In addition there were two infants who had been received into the European home, little twins who had been rescued from a living death. Their mother had died at their birth; the natives in this district on such occasions, having no means of nursing motherless bairns, are accustomed to bury the babies with the dead woman. In this case they were taken care of by a Catechumen and received at the mission station. Maw's feeding-bottle proved a great source of amusement to the older natives, who travelled many miles to see the way the children were fed. The native when first approached resents any treatment when sick, but after he has been treated successfully the news spreads rapidly along the coast, and others come and ask when we are going to their villages.

### An Out-Station.

It is now three years since I came into the mission. I was at once sent to an out-station, Mukana, with a layman, to take charge during the absence of the missionaries who left for England. The work here was new, and I had to assist generally with anything—teaching, laundry-work, or gardening. At that time there was an epidemic of influenza raging, and although one visited the sick daily, little could be done for the more severe cases of pneumonia, especially if the village (witch) doctor said they were going to die. After this all food would be refused, the patient's mats would be carried to the beach, and he would lie on them quietly until the spirit passed away. One is struck with this after a hospital life amongst our own people, who, fully awake to their sinful lives when they have to meet their Creator, are less willing to leave this life. In many cases the person who has bewitched the other is paid by the friends handsomely, the spell is taken off and the victim recovers. At the death there is a great deal of ritual, tribes from distant parts come to cry, and a large feast is given. Until we came they were burying under their dwelling-houses, but the Government is helping us to see this is not done—proper burial grounds being allotted. Polygamy, infanticide, and cannibalism are not yet out of practice, but these crimes vary very much in the different districts. No candidate is accepted for baptism indulging in polygamy, and cases of infanticide, reported to the District Resident Magistrate, are severely dealt with.

### The Need of More Help.

It is now rather more than a year since I gave my work up at Mukana. I then had the privilege of seeing our other stations, all badly needing more ladies and the visit of a medical man, as the layman in charge is likely to get all sorts of diseases amongst his people. As we passed through the villages we saw beri-beri, yaws, and leprous ulcers, in all forms. There are a large number of persons suffering from paralysis, acquired in many cases. On one occasion the clergyman and I insisted upon an old man standing up, then helped him to walk some distance; he had not left the ground for a very long time; his friends looking on were amazed, and were most indignant. Shortly afterwards he collected sufficient courage to walk to the mountain and visit the mission station.

### Crippled Lepers.

Persons suffering from leprosy almost isolate themselves from their families. Often one sees a tiny shelter built on to the general dwelling-house, and in this the leper lives, sitting on a raised platform with a large fire made under to well smoke his poor wounds, thus keeping flies and insects away. Here he remains unwashed, uncared for, until his poor limbs become malformed and crippled. On our way to our new work, my fellow-nurse and I found one almost glued to the platform; we went to the *Tau Bada* or chief of the village, and got his consent to carry her to the river, a short distance off, and to clean and dress her. Evidently she had not been moved from her right side for months. The only thing we could do for her was to show her how to make poultices with a native plant, and dress the wounds herself. We have heard since she is at least cleaner for our visit. But these cases can only be alleviated by our Mission, no measures, or help from the Government to segregate have been made, and our dispensary would need to be very well stocked to do half we would like to accomplish.

### A Hospital for White People.

I am now at Mambare River Station nearly two miles from the tiny township of Tamata, which consists of the Government station, gaol, and three stores. The goldfields are a day or two's walk from this township, the camps of the miners are very scattered, and I believe mining is carried on in a very primitive manner. Last year we opened a small hospital for white people, with a native one at the back; of which I have charge. Amongst the miners there has been a good deal of hæmoglobinuric fever, in many cases death having taken place before the Government carriers could bring them in. The other day a native boy was four days walking in; having blown his hand off with dynamite, he came here to have it seen to. As gangrene had set in I amputated the lower third. Our hospital is not yet properly equipped so I had to use a household saw. The wound has done very well notwithstanding.

### Beri-beri and Measles.

We have lately been visited with beri-beri and measles; both having been brought up from the coast by carriers, the latter from Cooktown. With the exception of our white staff, every person on this station was down with measles, and we lost one little girl; apparently the disease came to us in an exaggerated form here, pneumonia being very common. In some cases an attack of pemphigus immediately followed before desquamation had properly taken place, and the patients were fearfully reduced. I am pleased to hear that they are isolating beri-beri on an island off Samarai—our port—so that all cases occurring in this district will be sent there.

### Arrival of a Doctor.

At last the Government have secured a doctor for Samarai who is in charge of a neat little hospital. We had no qualified medical officer this side of British New Guinea until the Government appointed Dr. Hancock from England. It is hoped that he will do much to help those suffering from malarial and blackwater fever. At present I am in rather a fever-stricken district, the anopheles abounding at certain seasons; the rainfall for last year was 140 inches We are just getting into the rainy season again, when wettings have to be avoided, but if this is done, and quinine taken regularly, one is able to keep very good health. The nurses of the Sydney Hospital have just sent us a lovely battery, which was much needed, for neuritis, paralysis, etc., are most common. Let me ask any sisters in the profession, who have it in their power, to think of us in this far-away country when old instruments or hospital appliances are being thrown aside. Everything is useful in this remote region, I trust, too, that ere long we may welcome other nurses to this much-neglected colony.

### A Night-Duty Memory.

BY A NIGHT NURSE.

I was "night staff" in the male accident ward. I remember it had been a terribly full day, and two fresh cases had been admitted just before I went on duty, so I was as busy as I could be up to twelve o'clock. I had gone into the ward-kitchen to drink a cup of tea, which my probationer had poured out for me, when suddenly the accident bell went again. "Another case for us, Nurse, I am afraid," I said to my probationer as I left the kitchen, to be in readiness, and almost as I spoke, the porters and a policeman carried a patient in. My heart sank when I saw him. He was a young fellow about twenty-four, and apparently a gentleman's son. He must have been recently dining, for he was in evening dress, and there were miserable evidences that he had been drinking heavily. The account of the accident was that he had been knocked down by a passing vehicle, the wheels causing severe internal injuries. The head and face also were terribly injured, and one arm was broken. From the first the case seemed hopeless. A letter was found in his pocket, and I at once sent a message to the address on the envelope, hoping to find there some relative or friend who would come at once, for I felt there was no time to be lost.

In less than an hour, a lady, also in evening dress, with an opera cloak thrown over her shoulders, came, horrified and awe-struck, into a hospital ward for the first time in her life.

I went quickly forward to meet her, and asked if she were his mother. She bowed her head to assure me. I spoke to her as hopefully as I dared; she did not reply but followed me to the bed. I expected to see her bend and kiss him, to weep, or speak some sorrowful words; but as she looked down upon her son her face went deathly pale, and I saw her clasp her hands tightly together lest one should escape involuntarily and touch him. Then, turning from the bed, she signed to me to follow her, as though she wished to speak to me.

I led her into Sister's sitting-room, and with an expression of intense anguish she said, "Tell me, Nurse, did you find a gold watch and a signet ring upon my son's person when you undressed him?" I told her that no valuables of any kind were found upon him, and no money. She covered her face with her hands. "Ah," she cried, "this is cruel; three times this year my son has broken my heart! Three times have I gone in my own person and redeemed these possessions that I might screen him from his father. To-night, though I understand he may die, my heart will not break again—it is hard and cold—I feel no love, no pity, for to-day my son lied to me. I leave him to your care; do for him as you do for these others" . . . and she glanced through the open door, as she spoke, at my full beds. "He is dead to me already, for he has deceived me."

She was turning to go, when my probationer passed the door. I went out quickly, and in low tones told her to go quickly for "Night-Sister." I was determined not to let a mother leave the ward like that, and I knew if anyone could persuade her to remain it would be Sister Mary.

The latter came at once, and grasped the whole situation in a moment. I left them together and went back to my busy ward.

In a few moments I saw Sister in her bright, brisk way preparing some coffee, and presently she came out of the room, closing the door after her. "I must go, dear," she said, "I have such a long round before me. I think you will find it all right now; she will stay. Poor lady, poor mother! I am glad you sent for me; I can see you have a hard night before you, be sure to send again if there is anything I can do"—and with that she was gone. Dear Sister Mary! what a treasure she was to us all; always just the right one in the right place.

Towards three o'clock I was sitting by the poor boy, watching anxiously, for I thought I detected a slight sign of returning consciousness, when I felt a light touch on my shoulder, and looking up saw the pale face of his mother beside me. "May I sit here a little while?" she asked. I at once rose and gave her my place. "If you have other things to do will you come back very often?" I promised not to be away many minutes at a time; but I had scarcely commenced the many duties awaiting me, when she called me back. The boy was conscious, and asking for water; and as soon as I had given it him he turned and recognised his mother.

"This is 'goodbye,' Mother," he said in a weak voice. "I am going to die." I think she must have known it was true, for she knelt beside him and only said, "Sonny, Sonny!"

Then his voice came again a little more distinctly, while his eyes were fixed upon her face: "Can you promise I shall be forgiven the lie I told you to-day?" "Darling," she answered, "I do forgive you." "Yes, Mother," he whispered, "you, but God must forgive me before I die. I have no time now to make amends."

I wondered how she would answer him, and when the words came gently, but without hesitation, I knew she must have found some comfort in Sister's room that night. All hardness had vanished from her face, and before answering him she kissed him gently. "God bids us forgive, until seventy times seven," she said, "and He would not ask us to do a thing He could not do Himself." "You are sure it is all right, Mother," he asked; but I could see that speaking was becoming an effort. "Quite sure, my darling; try now and go to sleep. Have you much pain?" she asked a little later. "Not more than I can bear . . . and, Mother, give Father my love—tell him I had an accident, do—not—say—I was"—— Seeing she understood, he did not speak the hated word, and closing his eyes, drifted again into unconsciousness. They were his last words. He died about eight o'clock that same morning.

His mother's calmness was very wonderful. After I led her away from the bedside, she sat for a little while in Sister's room, waiting for a cab to take her home.

I went in to say "Good-bye" before I went off duty; and taking me by the hand, she said, "Nurse, do you think Sister would give me that little green paper?" and she pointed to "For Reading to the Sick" in The Hospital, lying on the table. "There are some words there that taught me what to say to my boy when he asked me that terrible question last night. I found them after your Night

A NIGHT DUTY MEMORY—*Continued.*

Sister left me with the book in my hand. Every day and night, so much must happen here which speaks to you all of another world; but in our life there is so little, so very very little"—— She could not speak any more. I took her to the cab, and she kissed me, and seeing my eyes, too, were full of tears, she pressed both my hands and said, "Bless you, dear, for your sympathy, I shall not forget you; you and your dear Night Sister will be in my mind whenever I think of my boy, and this one long, sad night in your hospital."

### Nursing in "A Haunted Ward."

BY AN OCCASIONAL CORRESPONDENT.

I had been at the hospital sixteen months when, one day at dinner, Matron came in and said, "I shall want a nurse to-night to go to "Lower A." with a case of suspected small-pox. Now, I am not going to ask any one of you to go, as the ward is rather isolated, but will any nurse volunteer?"

I saw the nurses look from one to the other, but no one spoke, till my colleague, who had only lately recovered from diphtheria, said she would go. But Matron replied, "Thank you, Nurse; I am sorry, but I could not think of allowing you to go, as you are still delicate."

Then she looked round her inquiringly. There were 24 of us, and none of the others spoke, so although I was only a junior in comparison with most of them, I rose in my seat, and said, "Shall I go, Matron?"

I fancied a peculiar expression passed across her face before she answered.

"Very well, Nurse. Come to my room when dinner is over."

When she had gone out and closed the door behind her, they all began talking, and I listened to scraps of conversation like the following:—

"Fancy expecting us to go down there with one solitary case!"

"I suppose you know you will be completely isolated"—turning to me—"until the case is properly diagnosed?"

"I don't mind particularly," I answered. "It will only be a matter of a few days, won't it?"

"Well, it depends; of course, if it is small-pox, you will have to stay there till the case is finished, and if I were you, I would ask for an increase of salary, but for my own part I would rather be content with what I have now than go down there, desolate, lost-looking place," this from a nurse who had nearly finished her three years.

It can easily be imagined that these assurances did not make me particularly cheerful, and matters were not improved by my overhearing a sister who was never very amiable towards me, sneeringly remark, "Oh! let her go, it will do her good! take some of the nonsense out of her, and perhaps she will not be so high and mighty when she comes back!"

Then my colleague who was sitting next me, whispered, "Take no notice of them, dear, perhaps you won't be there very long, and at any rate you will have an easy time with only one patient, unless of course it is a severe case. I wonder if it is a 'he' or a 'she'?"

### The Last Nurse in the Ward.

At length dinner was over, and I went to the matron's room.

"Nurse," she began, "I don't think you are much given to nervousness, and I am pleased with you for offering to go. The case is a woman who has been sent in from the General Hospital. She was admitted there with a compound fracture, and has developed a rash which has every symptom of small-pox; of course, it may or may not be. But she will need great care, as any delirious tossing may be very injurious to her broken limb."

She was busy sorting papers for me, and without looking up, asked if any of the nurses had said anything about the ward. "They don't seem to envy me much, Matron," I answered.

"Well, they are very foolish, because I do not really think there is any cause for fear. There are rats under the ward, which, as you know, is like all the others, raised from the ground, and movable, so if you hear any noises, it will be from them. But the nurses are very superstitious, because the last nurse in that ward on night duty went mad, and is at present in the asylum."

My face paled at this information, but Matron went on:

"I think she would have lost her reason in any case, as she had always been very peculiar in her manner, and I rather blamed myself for leaving her there six weeks, as the loneliness may have developed her malady sooner than otherwise, although she had five cases, but as they were foreigners, and could scarcely speak a word of English, they would not be much company."

She handed me the charts, etc., and then said she wished me to go and rest, as I had been on duty all the morning and was to be up all night. I left her feeling rather sorry that I had volunteered to go. When I reached my room, which was shared by two others, instead of resting, I began to collect the things which I wanted to take with me, and put away the rest in my box, as the room would doubtless be used by the nurse who took my place on day duty. Then I removed my cap (it was not worth while undressing, being already late in the afternoon), and threw myself on my bed, to try and sleep. I was disturbed at 7, had supper, and then Matron said she would accompany me to the ward. I was glad I had taken the precaution of bundling my belongings together, for she eyed them and said: "I see you have brought your things with you, nurse; that is right; as you will not be able to come up to your room very often, if it is small-pox, except when you are thoroughly disinfected." On our way we met some of the day nurses going up to supper, and they looked at me commiseratingly, until I felt as if I were going to my own funeral.

### A Ward Without Blinds.

As we neared the ward I remarked the absence of any blinds to the windows, and Matron said she was afraid I should have to be without them that night, as the ward having been empty for some time, and only short notice having been given that a case was coming, there had not been time to wash them and put them up, but they would be ready for the next night. The wardmaid was on duty with the case, as the nurse who was to do day duty would not be there till the following morning. She had been sent for from a nurses' home, which supplied the various hospitals with fever-trained nurses when they were short-handed. The patient was sleeping calmly, and was not apparently delirious, which was a great relief to me. Matron gave a few parting directions, and said she would be round again at 10 o'clock as usual. When she had left, the maid, who had been working in the laundry while the ward was unoccupied, turned to me and said:—"Well nurse, I have left everything as straight as I can, and I hope you will be comfortable enough, but it will be better to-morrow; there was not much time given to clean down before the case came." Then she too departed, saying as she went out: "I am sorry to leave you here alone, but I shall be almost as lonely as you are, nurse,"—for she, too, had to sleep in a small isolation adjoining the ward. I busied myself during the first part of the night putting the various things belonging to the ward into their places as they were in a hopeless muddle. The patient, beyond being disturbed a few times for medicine and nourishment, slept placidly, so my mind was easy that there was no delirium at present.

### The Doctor's Advice.

When the Doctor came, he shivered a little, as he walked up the ward, and said:—"It looks very eerie, nurse—where are the blinds?" I told him; he just shrugged his shoulders, and bade me keep up good fires. He stood by the woman's bed, and took her pulse and respirations, without awaking her; noted the temperature which I had already charted, then turning to me, said: "You will find it hard to keep awake, nurse, with only one case, and scarcely anything to do, at present, at all events. Have you anything to read?" I answered in the affirmative, and also said that I hoped I should not so far forget myself as to sleep while on night duty; that I never had done so. He laughed and rejoined: "No nurse, I know; but still you will find that there is not quite so much work to be done for one as for thirty." He turned to leave the ward, saying: "Well, she's all right so far, and if any change occurs, just ring me up. By the way," with his hand on the door knob, "have you heard the rats yet?" "No!" I answered. "Well," was his reply, "if I were you I would keep the doors fastened, and as some of the keys are missing, just balance a chair under the knob, and then the brutes could not come in and take you unawares." Matron had already been again, so I thought I would take his advice and fasten the doors as I did not wish to meet any of the nocturnal vermin. There were two wards comprising the whole known as "Lower A," divided by a small kitchen and a sitting-room, where the nurses had their lunch and tea. The door leading from the kitchen had a small porch, and it was through this entrance that anyone approaching the ward would probably come. They could, however, enter by other doors, as at the end of each ward was a bath-room, a tank-room for wet linen, and an isolation-room which held a bed and ward furniture sufficient for one case if necessary. Each of these rooms had a door leading out from it, so there were seven doors altogether, and the only one which had a key, was the kitchen door. I succeeded in balancing chairs under the knobs as the doctor had advised, and then stood in the kitchen looking up each ward. There were 12 beds in each, but the only bed made up was that in which the patient was sleeping, and as we had not received notice of any other patients coming in, there was no likelihood of them being made ready for use at present. The skeleton beds, showed dimly in the half light of two gas jets, and the glow of the firelight from the stove in the middle of the ward cast fitful shadows on the blindless windows, which looked out into fields on either side. I walked to one, and looked out around. I could see the lights from the nearest ward, which was five minutes' walk away, and I sighed to myself that I had not enough to do to pass the time. The clock in the sitting-room struck 2 a.m. It startled me somewhat, and I walked to the patient's bed and gave her her medicine, and she almost immediately sank into a doze again. I then thought I would make myself a cup of tea and read a little in the kitchen, where the lights were full on, and there was only one window, whereas there were three in the sitting-room.

### A Startling Surprise.

I had been reading about an hour, and could easily hear the patient breathing from where I sat, when I jumped to my feet in a hurry! My patient was out of bed! I could distinctly hear the pitter patter of bare feet on the floor! I rushed up the ward to find the woman still sound asleep in her bed as I had left her. I could not understand it. I was as wide awake as ever I had been in my life, and could have declared I had heard the tread of naked feet. I went and sat down again with my book; in ten minutes' time the same thing occurred again, and again I hurried to the ward, only to find myself again mistaken. Then I sat on the chair by the bedside, determined to wait and see what caused the noise. Half an hour passed, and there was still no sound but the regular breathing of the woman, and I thought I would go and read a little longer. I got up to go, and half way down the ward, stopped dead! The feet were walking slowly but certainly behind me—heavily, like those of a big person! I stood as if turned to ice! then, half fearfully, looked round, quite expecting to see the face of some visitant, but no, there was no one, and shivering, I returned to my seat by the bed. I only left it to attend to the woman once or twice, and to replenish the fires, until the grey dawn at 5 o'clock cheered me with the thought that day was near, and the echoing footsteps would cease. I busied myself then with washing the patient, making the bed, and various other things which need not have been done till nearer the time of the wardmaid coming on duty, which was 6.30 a.m. Presently I sat down with a cup of tea and meditated on the events of the previous night, and wondering whether I should tell Matron, and refuse to go on duty. She would probably tell me I was the victim of a hallucination, and it might prevent my promotion. Besides, things might be better the next night if the blinds were up. So I decided to say nothing that day.

### The Matron Inquires.

Before retiring to rest in the isolation room adjoining the ward—which was a tiny little place to accommodate three nurses, and which the day-nurse and I shared between us—I took a walk in the grounds surrounding the ward, and there met Matron coming to complete her round.

"Good morning, nurse," she said, cheerfully. "How is the patient?"

"Very comfortable, thank you, Matron, but I can't say so much for the nurse," I answered dubiously.

"What do you mean?" and she looked annoyed.

"Well, there are a funny lot of noises in the ward."

"Oh! nonsense, nurse, I told you that there were rats, and I intend to have them sulphured out to-day."

"That would be a good thing, but it was not rats I meant, Matron."

"What then?" sharply. I looked at her straight in the face and said:

"You might as well be frank with me, Matron; I am not nervous, and will stay in the ward as long as you choose to keep me there, but it is haunted, now is it not?"

"Nurse, I have heard such things said before, but I do not believe it, and you are only copying the others by saying so."

"Indeed," I answered, "the other nurses have not told me anything about it. We have never had occasion to mention "Lower A.," and besides I should not have paid any attention to it if they had, until I myself knew it to be true, but I do solemnly aver that the ward is visited between midnight and dawn, but only when the nurse leaves the patient's bedside, at all events, it was so last night."

"Well, nurse, if after being on duty another night, you still say the same thing, I myself will sit up all night with you, for unless I hear it with my own ears, I will not credit it." So she went on her way. I knew she would not believe me, and felt sorry I had broken my resolution of not telling her.

The Experience Repeated.

However, the next night was just the same as the previous one, notwithstanding the fact that the blinds had been put up, and the ward underneath had been sulphured, and nine large rats had been killed with sticks by the porters who were waiting for them, whilst dozens of smaller ones, who had been overcome by the fumes, were raked out afterwards. The following night Matron sat up with me, and was fully satisfied that what I had said was true. She did not even pooh-pooh my theory that at some time a patient had been neglected by a nurse, had got of bed, and perhaps died in consequence. She offered to let another nurse sit up with me for company, but I said I was not frightened, for so long as I did not attempt to leave the patient, I never heard the strange sounds. Moreover, as the patient's symptoms of small-pox were fading, there seemed to be a probability of my not having to be there much longer. So I begged Matron to say nothing to the nurses of what we knew, as it would be a surprise to them if I declared that I was all right in the ward. So I stayed till the patient was quite better of the rash, which was not small-pox after all, and she was transferred to an isolation in another ward. I was glad to return to my old ward on day duty and mix with the others again, a little sobered, perhaps, but quite unharmed by my short period in the haunted ward. A few months before I left the hospital to take a sister's post, it was necessary to make some alterations in the lower wards, and one of them having to be extended, some of the odd buildings were pulled down, "Lower A." being among them. That this was due to the Matron's instrumentality, and owing to the weird night she spent there with me, I shall always firmly believe.

### State Registration.

### MEETING AT CHELSEA INFIRMARY.

State Registration for trained nurses was the subject of a well-attended meeting of matrons and nurses, held at Chelsea Infirmary on Friday evening last week. The chair was taken by Miss Isla Stewart, Matron of St. Bartholomew's Hospital. Letters of regret were received from Sir James Joicey, M.P., Dr. Farquharson, M.P., Mr. Munro-Ferguson, M.P., Mr. William Jones, M.P., and from Mr. H. J. Tennant, M.P., who had been announced as one of the speakers.

Miss Stewart said that the subject of State Registration was as important as any that could be brought before nurses, and the meeting had been called in order that the arguments in its favour might be adduced, and that those present might make up their minds whether they would be what she described either as progressive or reactionary. She had been a nurse since 1879, and had always felt that the one thing needed was union at first her ideas had not extended beyond trade-unionism, but with experience of organisation and knowledge of nursing generally, they had crystalised into the opinion that State Registration was the one means by which nursing could be elevated to the rank of a profession, properly so-called. At present there was no profession, only a conglomeration of units, with no cohesion, no form, and no idea of self-government. She thought that the more thoughtful among hospital nurses, and a large number of private nurses, were in favour of registration; while on the opposition side were the employers of nurses, and others who had vested interests. She believed she was addressing a good many matrons of large infirmaries in London, and they would, no doubt, agree that one result of registration would be to create a certain amount of internal competition, which was very much needed. Many nurses nowadays aimed at getting through their training with as little trouble as possible, as a means to earning a living, and she thought, as the head of a large training school, that this was on the increase. The effect of registration would be to stimulate enthusiasm, which was not so marked a feature of modern nursing as of the days when the work was harder and opposition keener.

Lady Helen Munro Ferguson then summarised the arguments in favour of registration, remarking that one of the first things to be done by a central council, such as the promoters of the scheme desired to create, would be to decide upon the duration and quality of a sound, all-round hospital training. It could hardly be said, she averred, that any hospital had ever deliberately thought out a scientific scheme of training; this was largely controlled by the necessities of the patients and the financial resources of each institution, and was frequently left very much to chance, while even the leading Metropolitan hospitals were not agreed upon the subject. She believed that registration would strengthen the hands of the matrons, because they would have behind them a strong body of experts, forming a council on which they themselves would probably serve. The South African war had proved the disadvantage of there being no register of nurses from which supplies might be drawn, and the result of the war had been the re-organisation of the military nursing service. She maintained that organisation was equally necessary with regard to civil nursing, quoting the Queen Victoria Jubilee Institute as a body having a recognised period and standard of training, while for poor-law nurses in Scotland, registration was practically an accomplished fact She urged the nurses present to give their approval to the principle of State registration.

Mr. James Cantlie, F.R.C.S., proposed a resolution to the same effect. No one, he said, could define a well-trained nurse. Nursing was a branch of practical medicine, and it was degrading to a nurse to be obliged to court the approval of the individual doctor under whom she worked in order to gain a certificate. It was time that an independent standard of proficiency was created, to which it should be open for every nurse, wherever trained, to attain. The promoters of registration were anxious to have the opinions of nurses on the subject, and he urged them to let their views be known.

Mr. Richard Rigg, M.P., having outlined the course of Parliamentary procedure, and promised his support to the principle embodied in the Bills, seconded the resolution which was carried.

Miss Mollett, matron of the Royal South Hants Infirmary, and Mrs. Bedford Fenwick having spoken—the latter remarking that it was proposed to ask for a Select Committee of the House of Commons to inquire into the entire nursing question—the meeting terminated with votes of thanks.

### TRAVEL NOTES AND QUERIES.

By Our Travel Correspondent.

London to Switzerland (E. T.).—1. You give no pseudonym, but, I, hope you will see this. There is no sliding scale as to prices on foreign tickets; decide where you want to go, and send to Messrs. Cook, Tourist Agent, Ludgate Circus, E.C., enclosing stamped and addressed envelope. He will give you the exact price of the tickets required. 2. I am doubtful if you will get accommodation at such low terms as 4 francs per day in July, because that month is in the height of the season. However, write to the following places on a return foreign letter-card: Hôtel des Alpes, St. Beatenberg. This is above Lake Thun; their terms begin at 5 francs. Near to Mürren, above Lauterbrunnen, Pension Schilthorn, 5 francs. At Vevey, above the Lake of Geneva, Pension des Alpes, cheap, but I do not know their exact terms; also at St. Légier, three miles above Vevey, Pension Richemond, 5 francs. At Macolin, near to Bienne, there is the cheap Pension Magglingen. I have no personal knowledge of the place. 3. It is quite easy to forward small luggage by post. Your landlord will give you full particulars. There is no difficulty, and it is done at small cost.

### Midwifery: as Practised by Native Midwives in Fiji.

By the Hon. B. Glanvill Corney, Chief Medical Officer of the Colony.

A desire having been expressed that I would place before readers of the *Nursing Section* of The Hospital an outline of the practice of midwifery by native midwives in the islands where I reside, I have put together the following particulars, partly from my own observation, partly from the spoken testimony of numerous native wise-women of my acquaintance, and largely from materials I collected and recorded in association with Mr. Basil Thomson and the late Mr. James Stewart, C.M.G., my fellow-members of a Commission appointed by the Government to inquire into the causes of the decrease of the native population of the islands. The report of that Commission was published as a Colonial Blue-book in 1896 and fills a folio volume of 226 pages and four appendices, to which readers who wish for more detailed information on the general subject may refer, either at the library of the Colonial Office, or at the British Museum (8155, h. 12).

The disorders to which the women of such uncultured native races as the aboriginals of Fiji, who are hybrids or mixed descendants of the Polynesian and Melanesian peoples, are subject during pregnancy, are not devoid of professional as well as humanitarian interest; but the subject is too lengthy for treatment here. Some of their ailments are real, and some are fancied, according to native ignorance and prejudices. But it is safe to say that they are but little understood by the people themselves, and have only of late years been studied by European medical officers.

The same remark will apply to the process and the management of parturition as it occurs in the women of this interesting people. The key to all the practices of the native wise-women, or practitioners, is empiricism. Their medicine lore is banded down from mother to daughter, and in some cases from father to son. It is generally invested with an air of mystery: it is rarely in the nature of common sense, and it is seldom useful or successful except in the case of a few valuable herbs, barks, or leaves containing principles allied to some of those of the Pharmacopœia but prepared in the crudest manner and administered in bulky and very nauseous potions; and also in the application of massage, in which they are adepts.

Their midwifery is not more scientific than their general practice; its most redeeming feature is probably its prevailing policy of non-interference; while fortunately for the mothers, and also for the infants, the accidents of parturition, especially those brought about by malpresentations, are not of such common occurrence as is the case with European women.

With Fijians the approach of parturition is a signal for seclusion rather than publicity, although in the neighbouring islands of Tonga the reverse is the case. Only a few female relatives of the lying-in woman are admitted to the house when a Fijian mother is in labour, no such mixed and numerous attendance as is customary in Tonga, both within and without doors, being tolerated.

When the pains begin to make themselves felt a squatting position is usually assumed; but during the throes of childbirth a Fijian woman ordinarily resigns herself into a supine, semi-recumbent posture, the patient's mother or aunt and another intimate friend taking their stations behind her and supporting her shoulders, while the midwife plants herself in front, ready to receive the infant, for the whole affair is commonly of but short duration. Physiologically considered, this is, to English minds, a disadvantageous position for muscular action; but it appears to be adopted by chance, rather than by design. The woman occupies, of course, a place on the matted floor of the house—Fijians have no bedsteads, and raised sleeping platforms called *vata* (the equivalent of a dias) are only now being introduced, and that with difficulty. But in certain districts, where the climate is dry, and where superstition and the fear of witchcraft still reign with more force than mere custom, women, even nowadays, often repair to some hidden or secluded spot in the wilds, where they give birth alone and unaided, spending perhaps a night or two nights at the scene of the event in a small thatched hut, or even under a sheltering bush or clump of reeds, with nature's carpet for their bed-place; and then, after bathing in the nearest creek, trudge quietly homeward with their living burden on their back as if nothing particular had happened.

In Western Na Viti Levu, especially, it is a common thing for confinements (so to call them) to take place out of doors. In some instances a temporary hut, about 8 feet by 5 feet in dimensions and 4 or 5 feet high, constructed of reeds and grass thatch on a framework of mere sticks, is run up near the yam gardens, which are often situated at a great distance from the village. The pregnant woman takes up her quarters there with a cooking pot and a few odds and ends for the event, much as our domestic puss seeks out a retired nook in the garden, or in the family linen closet, or other (to her) convenient spot, and establishes herself there in a sort of nest, away from the turmoil of the kitchen and the maids' apartments. No mats are used on such occasions, but only grass or hay; as a rule there is no midwife in attendance or in waiting, nor even a grandmother; and the woman attends to her own delivery. No other preparation is made, but everything left to hazard. The people aver that they are accustomed to that sort of life, and prefer it: it being a usual practice for them to camp out in their yam patches for weeks during the planting season, tending the work. They then all live in one common shed by day, each family having its own temporary little hut for sleeping in at night.

The key to these primitive habits, in so far at least as parturition is concerned, is the belief in witchcraft. The object by whose agency a Fijian sorceress, or sorcerer, aims at signalising his intended victim is, as is usual with other savage races, some scrap of refuse such as hair, blood, food, and so on, from the person. Thus they are afraid to sleep with their heads on a *kali* (a wooden pillow) in those parts, lest some enemy should gather the stray hairs that may remain sticking to it and employ them as a medium for the exercise of witchcraft against their owner. They even abstain from corking up their water crocks, lest the water should be bewitched by the plug (made of leaves) falling into the hands of a sorcerer. And they never throw away the end of a *suluka*, or native cigarette, lest another should pick it up; but they stick it behind the ear, or in their bushy hair until a convenient opportunity presents itself for either burning it completely in a fire, or burying it secretly in the earth.

Hence the parturient woman has to face the difficulty of getting absolutely and entirely rid of the afterbirth and other impedimenta which accompany the entry of the child into the world, in such a manner that no evilly-disposed neighbour may gain possession of even a piece of soiled matting, or rag, and use it against her or against the offspring.

This superstition and the consequent practices are dying slowly before the march of European civilisation and teaching; but it is probable that the loss of comfort which they imposed upon a parturient woman was compensated by the strict degree of personal cleanliness and attention to detail which they engendered.

(To be continued.) p. 86

### Royal National Pension Fund for Nurses.

### ANNUAL MEETING.

The seventeenth annual general meeting of the members of the Royal National Pension Fund for Nurses was held at River Plate House, Finsbury Circus, on Thursday, April 21, 1904, "to receive the Council's report and statement of accounts, and to transact all other business appointed to be done at ordinary general meetings." The chair was taken by Mr. Thomas Bryant, F.R.C.S.

Supporting the Chairman were the following members of Council:—Mr. Edward Rawlings, Mr. Walter S. M. Burns, Mr. C. Eric Hambro, M.P., Dr. S. H. Habershon, the Hon. Egremont J. Mills, Mr. Charles W. Trotter, and Mr. Falconer L. Wallace. Amongst those present were Mr. Alfred J. Waley, Mr. George King, F.I.A., F.F.A., Dr. George W. Potter, Mr. Perceval A. Nairne (Chairman Seamen's Hospital, Greenwich), the Sister Superior, Nursing Sisters of St. John the Divine, Mrs. Pritchard Binnie (hon. secretary Junius S. Morgan Benevolent Fund), Mrs. Bretland Farmer (secretary Junius S. Morgan Benevolent Fund), Miss S. A. Swift (matron Guy's Hospital), Miss Oxford (lady superintendent Guy's Trained Nurses' Institution), Miss P. Peter (general superintendent Q.V.J.I.), and a very large number of policy-holders.

The Chairman, in opening the proceedings, said: I call upon the secretary to read the notice convening the meeting.

The Secretary (Mr. Louis H. M. Dick) having read the notice,

The Chairman said: Before I commence the business of the day, I must explain how it is that I find myself in this chair, for I am sure that it is a matter of regret to you all that the Chairman, Mr. Hambro, is not here. I am sorry to say that he is absent on account of illness. Sir Henry Burdett, our deputy-chairman, and the founder of this great institution, is also absent from the same cause. He is in the south of France, and you may be quite sure that it would be some great reason indeed which would induce him to be absent on such an occasion as our annual meeting. I am therefore, at the request of the other members of the council, taking the chair and conducting the proceedings to-day. We have received a good many letters of regret, among others from Mr. Thomas C. Dewey, Dr. Hawkins, and Sir E. Cooper Perry, who are members of the council, and who regret that they cannot be with us to-day. There are also many other letters in which the writers state why they cannot come, and express their regret at their inability to be present. I have now to propose that the balance-sheet and accounts, copies of which are in the hands of everyone present, be taken as read. I take it that that is the wish of the meeting, but if there are any of a contrary opinion, will they express it? Then I now rise to propose, "That the report, accounts, and balance-sheet be received and adopted."

### Continued and Increased Success.

I trust that you have all read the report, and have thought it over well. I myself studied it last night and the night before a good deal, and each page quite surprised me, because every page recorded continued and increased success. There is nothing in it whatever to produce any gloomy feeling or any feeling that the Fund is not carrying out to the utmost the purpose for which it was founded, and doing so, moreover, in a very astounding way. I do not propose to go through the report—I will leave it to your own judgment to go through it—but I wish to draw your attention to a few points which I consider of the greatest importance. The first point to which I will draw your attention is with reference to the new business, and you will see that 1903 was a record year because the Fund issued more policies than in any previous year since it was established, the number amounting to 953. This is all the more remarkable because there is no question that last year was a bad year from the nurses' point of view. I would point out also under this heading of increase of policies that while the number of policies taken out increased, the individual amount assured was somewhat smaller and the ages of the proposers were higher. These two hard facts tend to show that the older nurses begin to realise more and more the advantages of the Fund, and a great many of those who put off joining it were, in consequence of the want of work, brought face to face with the fact that, sooner or later, they would be at the end of their resources unless they started making some provision for themselves, even at a time when it was more difficult to do so than it had been for some years previously, for, of course, as age increases the premiums increase.

### A Point for Younger Nurses.

I would therefore especially draw the attention of the younger nurses to consider this point closely, so that they should not be in the same position as some of these later and older members. The decrease in the withdrawals is another point to which I desire to draw attention. It is a very striking feature, for with the expansion of any insurance business, one necessarily expects a larger number of withdrawals; but with this Fund such has not been the case, and it is rather noteworthy. As might have been expected, we paid away last year more in pensions than during any previous year. This, of course, is not to be wondered at, seeing that this side of the Fund's business must gradually increase, or its usefulness would cease, but the rate at which the increase has taken place is nevertheless very remarkable. The sick pay was again very heavy, and the result a net loss. The sickness fund, however, has now been strengthened, as explained in the report, sufficiently to meet all calls which are likely to fall upon it. That is a point to recognise, because some years ago there was some difficulty and doubt connected with the sickness fund. That, however, has all gone since, and you need not at all fear that any calamity is likely to occur. The expenses of management—an important point—are still this year, as during the past 10 years, considerably within our actuary's loading. Although the percentage rose $\frac{1}{2}$ per cent. during 1903, it is still at a very exceptionally low figure. With respect to the reserve fund, I am disposed to leave this in the able hands of Mr. Walter Burns, for I know that he would like to have a pleasant subject to bring before the meeting. I must say a word or two—I could not pass it over—about the Junius S. Morgan Benevolent Fund. The good work done by this fund is highly appreciated and we cannot be too grateful to the committee, presided over by Lady Rothschild, for their kind help. I must not forget, also, that for five years some anonymous donor has supplied the secretary's salary. That is very kind. We do not know who he or she may be, but we are very grateful for the fact. We now come to a most important point—the quinquennial valuation. The result of the valuation for the five years ended December 31, 1902, was published in the autumn of last year, and you will notice in the report that the amount distributed was two and a half times greater than at the previous quinquennium. On this subject, however, I would suggest that Mr. King, who is our actuary, should enlarge when he has the opportunity of speaking to you later on.

THE SURRENDER VALUE OF A POLICY.

There is one other point to which I must refer, and that is the alteration in the rule respecting the surrender value of the policies. This subject, in reality, should not come into the report at all, as the new rule only came into force on the first day of this year, but as it is referred to in the second paragraph of the report, and it is one which is of the very utmost importance, I hope you will allow me to say a few words about it. It is, moreover, a very pleasant point to dwell upon. When the Fund was established in 1887 the rule was that after two years, upon a nurse withdrawing from the Fund, she should receive back her premiums alone and without interest. Nothing was charged for working expenses. In 1891 this had been altered, and the decision was to allow 2½ per cent. compound interest upon the money paid in and to deduct 5 per cent. for expenses of administration, with the proviso that should the expenses amount to more than the interest allowed, a nurse should nevertheless receive back at least as much as she had paid in. That was the rule up to quite recently. Now comes the point I wish you to bear in mind. The Council, with the idea of still further improving the value of a Pension Fund policy, and in view of the extremely satisfactory result of the third quinquennial valuation, decided that in future, after a policy had been in force for seven years, no working expenses should be charged, whilst the 2½ per cent. compound interest is allowed, calculated from the day the first premium is paid. This makes the surrender value of a Pension Fund policy without any parallel in the insurance world.

MISS BRINTON'S PAMPHLET.

In a little pamphlet I have here, written by an inquiring policy-holder, Miss Brinton states that there is no deduction from a Pension Fund Policy after its holder has been seven years in the Fund; and she adds, "I may point out that if, instead, she had invested in a Post Office Annuity, and were equally obliged to withdraw, she would receive the exact amount she had put in, and no interest whatever, no matter how long she had been paying her premiums." I know that a Post Office Annuity was very popular with nurses some little time ago, and it was spread abroad that it was far better than the one which I trust you are all joining. I have read what Miss Brinton says, and I think, after looking at the question quite fairly, we are justified in saying that the alteration now made makes the surrender value of a Pension Fund policy without any parallel in the insurance world. I have nothing further to bring before you beyond saying a word in favour of this little pamphlet, which is admirably drawn up, and which has evidently been written by an inquiring Pension Fund policyholder. I trust that it will be printed and circulated wherever possible. I do not think I shall have to urge Mr. Dick much to induce him to carry out this wish very fully. I now propose, "That the report, accounts, and balance sheet be received and adopted," and I will call upon Mr. Walter Burns to second that.

THE ELOQUENCE OF FIGURES.

Mr. Walter S. M. Burns: Ladies and gentlemen, there remains very little for me to say after the speech of your Chairman, who has put the whole position of the Fund before you. I wish first to associate myself with the great regret which has been expressed by him at the absence of Mr. Hambro (whose past services to the Fund since its very beginning are so well known to everyone connected with it) and of Sir Henry Burdett. I do not think that anyone connected with the Fund can look at its past history without a sense of the greatest satisfaction. The Fund was started in 1887 with a capital of £20,000, and at the end of 1903, as shown in the last year's report, the invested capital, without including the Junius S. Morgan Benevolent Fund, amounted to over £800,000. These figures, I think, must speak with far greater eloquence than anyone can as to the success which the Fund has had with the nurses, and I feel confident that the reason for this success (it has often been stated at this meeting, but it cannot be stated too often) is that the nurses realise that the Fund exists entirely for their benefit. There are no shareholders to consider and no directors to pay. The result is that the Fund can afford to give the nurses such terms as no insurance or other society can possibly afford to give. Other societies have to think of their shareholders and to pay their directors, and they cannot possibly give anything like the terms this Fund can afford to give. Further, we have the great advantage of having on the Council two men like Mr. Hambro and Sir Henry Burdett, men of the highest reputation in the City, and who ungrudgingly give their services to the Fund for nothing. No other fund in the world in this sort of business can say that they have such an advantage as that. I also wish to say one word before concluding about the services which are rendered to the Fund by the staff. We on the Council know with what unflagging industry and intelligence the staff carry on their work; and I can assure everyone that it is in no small degree due to their efforts that the Fund is in its existing state of efficiency and success. Ladies and gentlemen, I beg to second the motion.

£810,000 IN INVESTMENTS.

Mr. Alfred J. Waley: Before the resolution is put to the meeting I should like to say a few words. My reason for asking to speak is that I should like to make a few remarks with reference to the investments comprising the Fund to which Mr. Burns has referred. At the end of the year the cost price of these investments was £810,000, and it was my pleasure—as I have had the privilege for the last few years—to make a valuation of the securities comprising the fund. Taking the most conservative valuation possible (that is to say, valuing the whole of the securities at the price at which, in my opinion, they could be readily sold for cash), there was, in spite of the enormous depreciation that has taken place in securities during the last two years, quite a small depreciation in the Fund's investments. If the medium quotation of the day had been taken, there would have been a nominal profit on the cost price of about £1,000; but, taking the conservative valuation to which I have referred, the depreciation was only £2,500. I think the nurses must therefore recognise the ability and discretion which have been displayed with respect to the investment of their funds by those who have control of them, and they must feel that every attention is given to the security of the investments, while every effort is made to obtain the highest rate of interest possible. Perhaps I should say that the Junius S. Morgan Benevolent Fund shows an appreciation of over £1,000, and when we come to remember that the depreciation in securities has not been confined to this country, but has also prevailed in a very marked degree on the other side of the Atlantic, this fact shows the care and skill which have been devoted to this Fund.

The Chairman: We are, I am sure, very much obliged to Mr. Waley for his very interesting remarks. I will now put the motion. Those in favour of it will please to signify the same by holding up their hands. I will now put the contrary. I do not see anyone voting to the contrary.

The election of representatives of the policyholders was afterwards proceeded with, and Mr. Falconer L. Wallace and Dr. George W. Potter were appointed scrutineers.

The Chairman: We now come to the resolution for the re-election of the retiring members of the Council, and I will call upon Mr. King to address you.

Mr. George King, F.I.A, F.F.A.: Mr. Chairman, ladies, and

### ROYAL NATIONAL PENSION FUND FOR NURSES—*Continued.*

gentlemen, it is a great pleasure to me to have this resolution placed in my hands. The retiring members of the Council are our Chairman, whose absence to-day we all deplore; Mr. Everard A. Hambro, Dr. Habershon, Sir E. Cooper Perry, Mr. Charles W. Trotter, and Mr. Falconer L. Wallace. Some of these names are household words in the financial world, and others are household words in the medical world, and in an institution of this kind the co-operation of these two branches is most important. Our Chairman is well known among us, and his services are so highly appreciated that I need say no more about him. The other members are also well known among us; and as regards the medical gentlemen, we have through them the influence of our great hospitals brought to our aid. I have much pleasure, therefore, in proposing that these gentlemen be re-elected.

### The Safety of the Fund.

In moving the resolution, I will accept the Chairman's kind invitation to say a few words' principally with respect to the valuation of the Fund. This is the first occasion on which it has been possible to discuss this matter at one of the meetings, because the labour of the valuation is enormous, and the results on the last occasion were not ready. You, however, have had the main points in the valuation brought out in the communications sent out to the members, and therefore it is not necessary that I should enlarge upon them; but I should like to say this—that the one idea in the valuation and in the whole conduct of this Fund is safety. The Council look after the investments and make them safe, and whenever there is the least suspicion of even a small depreciation they take steps to put it right. As regards the valuation, it is also my object, acting under the direction of the Council, to make it thoroughly safe. There is no effort to bring out a surplus, but, on the contrary, there is every effort to keep large funds in hand so as to make the Pension Fund sure. The tables used are tables which give strong reserves, and the rate of interest brought into account is considerably less than that which is at present realisable, and there is therefore a certain surplus which in the future, when it has been turned into cash, will fall into the Bonus Fund. Five years ago there was a comparatively small surplus, but this time the surplus is a great deal larger. That does not necessarily mean that the bonus to individual members is larger, because there are more members to share it; but it means that there is at least the same margin of safety, because before anything could happen to the annuities the surplus would have to disappear. We have therefore a substantial margin of safety in the methods adopted by the Fund. We have in the Annuity Fund alone at the present moment investments amounting to nearly £714,000, and there is no question, I think, that when the next valuation, five years hence, comes to be discussed we shall find that we have at least £1,000,000. That is a splendid position. And remember that these funds of which I am speaking are not benevolent funds subscribed by philanthropic donors, but are the accumulations of the savings of the nurses themselves. That shows, I think, a splendid result; it shows how thrifty the nurses are, and that it is only necessary to have a thorough organisation to bring that thrift out and make it productive.

### The Certainty of the Annuities.

I have spoken of one certainty in connection with the Fund, but the valuation brought out another certainty—that is, that the nurses will live to draw their annuities. (Laughter.) It is not wasted labour for them to pay in, because they will certainly draw out. The rate of mortality among the members of the Fund is extraordinarily low. I see it stated in the report—not exactly in my words—that I had treated the nurses as *average* ladies, but I should like to make a small correction. Had they been average ladies the number of deaths would have been greater, but I did not compare the mortality of the members of this Fund with average mortality. I compared it with the mortality of select classes. The Government annuitants show comparatively light mortality, but the mortality in connection with this Fund is smaller. I compared it also with the select mortality of British offices, whose tables show a lighter mortality still than the Government annuitants, and yet the mortality in those offices is higher than among the members of this Fund. My comparison, therefore, is with select lives and not average lives. There is therefore this second certainty of this Fund — that the members will live to draw their annuities. In the speech of the Chairman reference was made to the rising rate of the expenses, but I should like to point out that that is inevitable—not that the rate of expenses is really increasing, but when annuities fall into possession the premiums cease to be payable on them. The expenses, therefore, have not the same amount of premium income to fall back upon, and yet they go on because it costs money to make the annuity payments and to carry on all the correspondence which the annuitants necessitate. There is no real rise in the costs of management; it is only an apparent rise on account of the nature of the working of the Fund itself.

### The Vitality of the Members.

There is one branch of the Fund which, to my mind, is not quite so satisfactory as the annuity branch—that is the sickness branch. We have had on two occasions difficulties over this branch, not risk—because the Fund is so strong that no possible calamity to the sickness branch could affect it—but the claims have been very abnormal. Here, again, the vitality of the members is most remarkable—more so, even, than among the annuitants. It is very strange that while our sickness claims are very much above the sickness claims of the female members of registered friendly societies, where the members are of a much lower rank of life, and therefore, one would think, more liable to illness, and while the sickness claims of the Pension Fund for Nurses are higher than the utmost sickness claims I know of shown by any table, yet the mortality is most extraordinarily low. I find in my valuation report that the number of deaths in the sickness branch was only 21, whereas those expected by the tables on which the original calculations were based was 71—21 against 71—and by the Government friendly society tables to which I have referred the expected death claims were 69, by the Government annuity tables 70, and by the British Office tables 66. Therefore we have only 21 deaths among the members against the lowest estimate of the other tables, 66, or less than one-third. Yet, as I have said, the sickness is very heavy. That is a very curious result, and I cannot altogether account for it. It comes to this, that if any nurse wants immortality she must become a member of the sickness branch and then fall ill, but she will never die. (Laughter.) This is a feature which is not to the benefit of the Sickness Fund, although I congratulate the nurses on the fact that there should be this very low mortality. The Sickness Fund would be better off, however, if the nurses would only condescend to die, as they could not draw sick pay after death. This very low rate of mortality is to the Sickness Fund a very great disadvantage, though I congratulate the ladies themselves on it. We have raised the rates on two occasions, but I very much regret to see that in the present annual accounts there does not seem to be any improvement in the course of this fund, and it will not be my fault, but that of the ladies themselves, if, five years hence, we have to raise the rate again. I will only add, in conclusion, that the Pension Fund for Nurses is in a splendid condition, and, as Mr. Burns has told us, and as I have over and over again stated in reply to inquiries, there is no institution in the world that can give benefits equal to those which this Fund provides for nurses, and it would be much to the advantage of the nurses if they would induce their friends to join it and share in its success. I now propose the resolution for the re-election of the retiring members of the Council.

Mr. BURNS: I second that.

The CHAIRMAN put the motion in the usual way and, having declared it carried, said: The next resolution relates to the re-election of the retiring auditors, and it is a pleasure which devolves on the Chairman to propose the re-election of Messrs. Whinney, Smith, and Whinney. It is arduous work to undertake, but they always do do it, and they have done it very satisfactorily.

Dr. S. H. Habershon: I am very happy to second the motion.

The Chairman: Before I put it, I will include with it the next suggestion I have on the paper before me—that is, that they be not only re-elected, but that their remuneration be 50 guineas. I now put that.

The motion was carried.

The Chairman: The result of the ballot for the election of representatives of the policyholders has just been handed to me, and it is as follows:—We hereby certify that we have examined the ballot cards received for the voting of the annuitants' and policyholders' representatives. One thousand four hundred and thirty-three cards were received, three were spoiled or contained more than eight names. The following is the result of the voting:—Miss Mabel Cave, 1,332; Miss E. Fisher, 1,320; Miss K. H. Monk, 1,325; Miss P. Peter, 1,329; Miss Florence Smedley, 1,313; Miss S. A. Swift, 1,332; Miss E. Vincent, 1,313; Miss Sophie Morris, 1,327; Miss F. M. Calvert, 5; Miss J. E. Styring, 4; Miss Agnes Watt, 4; two ladies, 3 each; one lady, 2 votes; 16 ladies, 1 each. I now declare that the first eight ladies are elected.

The ballot cards we must now order, if you please, to be entirely destroyed. You will undertake to see to that, Mr. Dick?

The Secretary: Yes, sir.

The Chairman: I am quite sure that we all thank the scrutineers very much for their hard work. I think that this concludes the business of our meeting.

Mr. Edward Rawlings: Permit me to propose a hearty vote of thanks to our Chairman for presiding over our meeting to-day. We are all very fond of Mr. Bryant, who is one of the original members of the Council, and I may say that I pride myself on being another. It devolves on me—and it is a very great pleasure indeed—to move a vote of thanks to the Chairman for presiding over our meeting on this occasion—a duty which he has discharged so pleasantly.

Mr. P. A. Nairne: I second that with very great pleasure.

Mr. Rawlings put the motion to the meeting, and it was carried unanimously.

The Chairman: I will not occupy your time, ladies and gentlemen, more than to say that I am very pleased to find myself in this position, because, as Mr. Rawlings stated, I am one of the original members of the Council. I confess, as I have stated before, that at the outset I said. If you think I can do any good I will join it, but I do not believe it is going to be a success." The fact is I had not such faith in the nurses as time has proved that I ought to have had. As time has gone on the success of the Fund has proved what a body of hard-working people can do by putting their shoulders to the wheel. It has been a splendid success, and it is a pleasure to me to do anything in my power to help it on. (Cheers.)

The proceedings then terminated.

### British Nurses' Association Conversazione.

ON Tuesday evening a Conversazione for the members and friends of the Royal British Nurses Association was held at the Portman Rooms. Between eight and nine the honorary officers, Mrs. Latter, Mr. John Langton, and Dr. Comyns Berkeley, were very busy receiving their guests, who arrived in such numbers as to throng the rooms. In an alcove in the central chamber the band of the Grenadier Guards was established and played a delightful selection of music throughout the evening, which was much appreciated. Excellent refreshments were supplied at a buffet in one of the outer rooms. At half-past nine Princess Christian arrived. Her Royal Highness, who was received by the honorary officers of the Association, was attended by Miss Locke and Major Evan Martin. A bouquet was presented by the daughter of Sir Anderson Critchett, which, being all made of white flowers, formed a pleasing contrast to the Princess's dress of black tulle covered with sequins. Many nurses and others had the honour of being presented to Princess Christian, after which she made a tour of the rooms, frequently stopping to speak when she caught sight of a well-known face. She left after a visit of over three-quarters of an hour. Among the company present were Sir William and Lady Church, Sir William Taylor, Sir Thomas Smith, Sir William Broadbent, Sir William Bennett, Miss Wilson, Miss Dalrymple Hay, Miss Peter, Sir R. M. Hensley, Sir Anderson and Lady Critchett, Mr. Louis Dick, Dr. and Mrs. Shuttleworth, and many others.

### The Trained Nurses' Annuity Fund.

A MEETING on behalf of the Trained Nurses' Annuity Fund was held, by kind permission of Emily Lady Ampthill at 19 Stratford Place, W., on Wednesday afternoon. The chair was taken by Lady Loch, one of the Patronesses of the Fund. There was a good attendance, among those present being the Dowager Marchioness of Dufferin and Ava, the Dowager Countess of Lytton, the Dowager Lady Ampthill, Lady Elizabeth Biddulph, and others.

A letter having been read from Princess Christian regretting her inability to be present and assuring the meeting of her sympathy and interest, Mrs. Garrett Anderson, M.D., urged the claims of the Fund, and suggested that patients, as well as nurses, should contribute to it. It was undoubtedly the duty of nurses to save, and their earnings should enable them to do so. Owing to the limited scope of the Society, there was no danger of the spirit of independence being destroyed.

Lady Helen Munro Ferguson, Miss Barton, and Miss C. J. Wood enforced the need for a benevolent fund, but differed from the first speaker as to the amount a nurse was able to earn, even when there were no slack times; and it was pointed out by Miss Barton that a nurse spent on her ward and patient, especially the children, money which, from an economic point of view, she ought to save for her own old age or sickness.

Lady Biddulph thought that the condition that an assistant should pay £15 to the Fund on receipt of an annuity of £17 was a hard one; to which Dr. Ogier Ward, hon. secretary, replied that the matter was receiving the consideration of the committee and was being modified. Another speaker remarked that the Fund helped a class of nurses to whom the benefits of the Royal National Pension Fund for Nurses were unavailable, since they had not been able to begin to subscribe to that excellent Society in time to participate in its advantages.

### A Visit to an Ambulance Station in Florence.

In a very unpretending looking house in the Via Ghibellina, Florence, is carried on a very useful and important work. The style and title of this department of hospital work is the "Ambulatorio Policlinico Regina Elena," and here in a quiet way are treated considerably over 2,000 patients every year. Every kind of surgical case which is not serious enough to be detained in a hospital is brought here, consequently a great variety of cases are constantly under treatment, and the nurses obtain a very good insight into surgical work. At the end of a long corridor is a large apartment well lighted and ventilated, and fitted up with all sorts of surgical appliances. An excellent operating table, which also serves as an examination table, stands on one side, and a high reclining chair on the other, both constructed as lightly as possible.

A part of the work done by this most deserving charity is the treatment of women's diseases, and apparently the director himself is at the head of this department. Two nurses are always in attendance, the senior of whom was trained in a similar institution in Rome, and is paid for her services, while the junior is a voluntary worker. They were both attired in the usual long white pinafore, and seemed to be thoroughly interested in their work. Upon the wall hung an excellent portrait of Queen Elena, who it is to be hoped will continue to show the same interest in charitable work as her august mother-in-law, Queen Margherita. Upstairs are several smaller rooms, one set apart for ophthalmic cases, another for the treatment of nervous diseases by the electric battery, massage, etc.; the director who kindly showed me round incidentally remarked that in his opinion, powder was much better than oil, and was always used by his nurses when rubbing their patients.

An English nurse who had spent two years in this institution had evidently left a good impression behind her, as the director spoke in the highest terms of her energy and capability, and of course I was pleased to hear that my countrywoman had been so much appreciated. Before leaving I was shown a portable weighing machine for infants, exceedingly light and easily taken to pieces, the scale (or seat) was made of strong coarse linen, and the whole of it could be easily carried in a district nurse's bag. It was the invention of the director who had obtained a patent for it. I also saw a special bed for accouchement cases, which was being exhibited at a neighbouring instrument maker's. Twelve doctors are attached to the staff of this institution and the patients receive treatment gratis. During a conversation with the chief doctor, I learned that at least some of the nurses have after a course of training received a diploma, but as it was impossible to secure the services of educated women as in England, it was necessary to employ the only class of women that offered themselves.

### Everybody's Opinion.

### PORTRAITS AS PRESENTATIONS.

"Army Nurse" writes: May I put in a word for military hospitals? As Lord Roberts is retiring from active service, this would be a suitable time to have his miniature painted. Nowhere would it be more appreciated than at Netley or Woolwich. Why should we not, by degrees, get a most interesting collection of great generals?

### A WARNING TO MATRONS OF PRIVATE NURSING INSTITUTIONS.

"Indignant Nurse" writes: I have read the note "A Warning to Matrons of Private Nursing Institutions." I also as a nurse can give my experience of a so-called Nurses and Nursing Institution in Lancashire. I may state that I have been private nurse for eight years in different parts of England, but the Home in Lancashire I refer to "takes the cake," as the vulgar saying is. The food was badly cooked and not fit to eat. Patients whom I have known complained and were removed by their relations, as they were in no sense of the word properly cared for, although paying good fees. I now take my own cases, not having been foolish enough to sign a paper. There are usually two sides to all questions. I know some nurses are too dainty to rough it, and matrons are sometimes to blame for their treatment of their nurses.

### FILLING A WATER-BED.

"Nurse H." writes: I should be glad to know the opinions of some of your readers as to the best method of filling a water-bed and placing the patient upon it in this particular case. The patient, a sufferer from paralysis, is very heavy and in very weak condition, on a wide bedstead and feather bed; no other bed or couch available owing to the small size of the room. No bath-room in the house.

Nurse Eva Torr, Altrincham, writes: I should very much like to give my experience. I was trained at the Salford Union Infirmary and have a three years' certificate. The way we were trained to fill water-beds was to fill the water-bed on a spare bed, drawn next to the patient it was intended for. When filled, we had to cover the water-bed with blanket and sheet, and push the bed quite close to patient's bed. Then gently we had to draw the patient by the sheet he was lying in on to the newly filled water-bed.

"Hope" writes: This question has appealed to me, as I have had many cases where a water-bed was essential. I have never had the good fortune to find a patient on a bed suitable to receive the weight of a water-bed. Hence when it has been ordered, I have also asked for a single bed, and for boards to cover the entire under mattress or iron laths, as the case may be. The ordinary mattress is placed on that, followed by water-bed, which I then have filled with warm water, and make up the bed as far as possible for the patient, who is lifted on with, I think, less fatigue than if he or she had been on the bed during the filling process. From personal experience I do not know of a more comfortable way. Of course when the patient is moved there must be really good assistance, and the sheet or blanket patient is lifted in must be very firm. But if this is so, I do not hesitate to say the result will be satisfactory.

### DOUCHING IN PRIVATE NURSING.

"Nurse S." writes: It has occurred to me that it might be useful to nurses in private practice to hear of a little contrivance in the use of the douche, which I have used successfully, and which, as far as I am aware, is not known to the nursing world. When copious, continued douching is required, the bed-pan, used in the ordinary way, is insufficient, as it requires to be frequently emptied. The use of a mackintosh, also, which is sometimes arranged instead, for drainage, has its drawbacks too, as the patient must then be drawn to the edge of the bed, which in the case of weak or helpless patients may be a difficulty, and which also causes more or less exposure. For my plan, all that is necessary in addition to the usual douche can and slipper bed-pan, are from one and a half to two yards of indiarubber tubing (which is quite cheap, and can be had from any good chemist), and an ordinary enema syringe. The tubing should be about the same thickness as that of the syringe. The water for the douche is prepared in ewers at the proper temperature, and the patient is made comfortable on her back, with the bed-pan under her. One end of the tubing is then laid flat on the bottom of the bed-pan inside, and loosely secured in position with a piece of tape. The tubing falls over the bed, and the other end is placed in a foot bath by the side of the bed, and the metal end of the enema syringe fitted on to it. The douching is then proceeded with in the usual way, and when the bed-pan is partly filled, the air is expressed from the tubing by means of the bulb of the syringe, when the water immediately flows into the bath and will continue to do so as long as any water remains in the bed-pan. The douche can is kept frequently replenished from the ewers, and with a little care it is easy to arrange that the inflow to the bed-pan, and the out-flow to the bath, be about the same and so to avoid any overflow into the bed. I may add that for the disinfection of the rubber tubing and enema syringe, in addition to the usual mode of cleansing, they may be boiled, and thus rendered perfectly sterile. I am indebted to a doctor under whom I have worked for this latter information. I have tried it, putting the rubber into cold water and slowly bringing it to the boil, only being careful to see that all air was first expelled, and I have found that the rubber did not suffer in the least from this treatment.

### STATE REGISTRATION.

Miss Annie R. Wilson, Lady Superintendent of the District Nurses' Institution, Cambridge, writes: I should like my name added to the manifesto against the State registration of nurses, as I feel that three years' training in any hospital is not all that is required for the making of a good nurse. There are some fully-trained nurses I know who are anything but a credit to the profession, and if such were upheld and protected by State registration there would be more harm than good done. If a woman doesn't naturally possess the essential qualities of a good nurse, no amount of training will produce it in her. Nurses are born, not made! Hospital training develops and completes what Nature has begun, and, in my humble opinion, a nurse—whether attached to an institution or working independently—ought to realise that her success in life should depend more upon her merits as a nurse than upon the "parchment" she holds.

Miss Helen Hunter, Matron of the Royal City of Dublin Hospital, Dublin, writes: The question of State Registration of nurses is, as the result of a meeting lately held in Dublin on that subject, so much under discussion at the present moment that I venture to submit the following remarks as presenting one or two aspects of the question which appear to me to have been so far inadequately recognised in such discussion. Registration is proposed as a remedy for certain abuses against which nearly every profession finds it necessary to protect itself. There is at present nothing to prevent any woman, however unqualified either by character, training, or experience, from calling herself a nurse—getting herself accepted as such, and thus forcing her own way into the ranks of a profession which she thereby brings into discredit with the public. Registration is proposed as a protection to the public from such imposture, as well as a protection to the credit of the other duly-qualified members of the nursing profession. It is not, however, obvious, that as regards the former point, protection of the public, registration would really act as a sound or effective measure. It is said that the unqualified nurse, having no certificate to go upon and nothing to show for herself, would not then be accepted by the public. But as the unqualified nurse has at present no certificate to go upon and nothing to show for herself and yet is accepted by the public, the finality of such an argument is still open to question. There would after registration be nothing more to prevent anyone from engaging an unqualified nurse than there is at the present moment. It is said that without registration the public has no means of determining the claims or status of any professional nurse; that with registration these claims and this status will be determined and fixed by the State, through the medium of competent judges and authorities, and that the individual engaging the nurse takes no risk. On the contrary, I wish to point out that this individual will take a good deal more risk. The nurse in question is registered, say, in 1904; anyone engaging her in 1914 takes all the risk of possible deterioration in personal or professional character or qualification during the intervening 10 years. Once registered the nurse herself is safe for ever, but the public has no such security as regards the permanent value of her services. In 1904 she may have been strictly sober and her character in every way admirable; ten years later this may be no longer the case. In 1904 her technical knowledge may have been up-to-date and her work careful and reliable; in 1914 what guarantee has the public that it is still equally so? To the public the register will always represent a sort of permanent safeguard—a guarantee that the nurse is a safe person to engage as long as her name is on the register. How far is this likely to be the case? Whose business would it be to take her name off the register should her work or character have seriously deteriorated? It is most unlikely that any individual suffering from such deterioration would undertake the inquiries necessary to effect this—what would be gained even by success in such an unpleasant task?—while it is still less likely that her own training school would go out of its way to bring discredit upon itself. The public must also be considered in the question of the expense of the additional training required by registration. By whom is it intended that this additional burden is to be borne? The money subscribed by the public towards the maintenance of hospitals is not intended for providing the hospital nurse with an education, the duration and extent of which is to be determined by the professional needs and status of the nurse herself. This difficulty can only be met by a raised entrance fee for the probationer, in addition to the fees for State examinations and registration. Practical experience in the training of nurses will also be found to show that two years' training in hospital is a better preparation for private nursing than a course of three or four years would ever be. Too many years of subjective hospital discipline and routine are no ideal preparation for the private nurse. The qualities most needed by her, the power of adapting herself to a variety of conditions and circumstances—these are not those best developed by many years' work in one general hospital and many years' habit of submission to unvarying routine and custom. Additional technical knowledge is bought too dearly, if bought at the sacrifice of that power of adaptability which is all essential in private nursing. Indeed, any system of registration by which mere length of training is made of undue importance, and by which the technical qualities of a nurse, rather than her personal ones, are made the chief basis of her right to practise, seem to me fatal to the best interests of the nursing profession, and calculated to place mediocrity at once upon a level with moral excellence while leaving the public no means whatever of judging or choosing between the two. Strength of character, devotion to duty, sound temper and judgment, power of endurance and of self-forgetfulness, these seem to me the materials of which, and of which alone, good nurses are fashioned. How is this standard expressed or even touched in a system of registration by which, granted the same course of training and the same duration of that course—the same hallmark is afforded, indifferently, to every woman that passes through it—to the best and the worst alike. Such a standard is too false to life to be of any advantage to the best interests of a profession in which the personal elements and personal qualifications must always remain so infinitely important.

### Appointments.

Colne and Holme New Joint Isolation Hospital, Maltham, near Huddersfield.—Miss A. M. Baker has been appointed matron. She was trained at Monsall Fever Hospital, Manchester, and the General Infirmary, Leeds. She has since been charge nurse at Brook Fever Hospital, London, sister of children's ward at Bolton Infirmary, sister of male medical and surgical wards at Cardiff Infirmary, sister of female surgical ward at the General Hospital, Birmingham, matron of Bury Fever Hospital, and matron at Hailey Sanatorium, Wallingford.

Darlington Fever Hospital.—Miss C. E. J. Chaffer has been appointed matron. She was trained at the Queen's Hospital, Birmingham, and has since been nurse-in-charge at Colchester Isolation Hospital.

Guest Hospital, Dudley.—Miss Ethel Parkes has been appointed night sister. She was trained at the Walsall and District Hospital, Walsall, for three years, and has since been doing private nursing on the staff of the Staffordshire Institution for Nurses, Stoke-on-Trent.

Hastings Union Infirmary.—Miss Florence Gray has been appointed assistant nurse. She was trained at St. Peter's Home, Kilburn, by the Meath Workhouse Nursing Association.

Newport Infirmary, Isle of Wight.—Miss Hilda Lea has been appointed superintendent nurse. She was trained at Brownlow Hill Infirmary, Liverpool. She has since been superintendent nurse at Lincoln Union Infirmary, and has also been attached to the private staff at the Royal Berkshire Hospital, Reading.

Sanatorium for Infectious Diseases, Scarborough.—Miss Kate Dunnill has been appointed charge nurse. She was trained at the Sheffield Royal Hospital and the Carlisle Fever Hospital. She has also been charge-nurse at Hungerford Union Infirmary.

### Presentations.

City Hospital, Walkergate, Newcastle-upon-Tyne.—Miss Mary Wynne, on resigning her appointment as charge nurse in the City Hospital, Newcastle-upon-Tyne, which she has held for five and a half years, has been presented with a gold watch as a token of esteem from the hospital staff. Nurse Wynne takes with her the good wishes of all her late colleagues.

Garstang Parish.—Miss Frances Dell, who is levaing Garstang, has been presented with a gold watch and chain, also an illuminated address and a purse of gold, in recognition of her services. The inscription to the address testified to the excellent work done by Nurse Dell for the parish.

Nottingham and Notts Private Nursing Association.—Miss K. Steen, who has resigned her appointment as matron of the Nottingham and Notts Private Nursing Association in order to take up the duties of lady superintendent of the Kent and Canterbury Nursing Institute, has been presented with a handsome barometer by the nursing staff, as a mark of their regard.

### Novelties for Nurses.

By Our Shopping Correspondent.

### A CHARMING PICTURE.

A most charming gravure is being offered by the Bovril Company in return for a certain number of coupons, of which one is enclosed with each bottle of Bovril. The gravure is after a painting which appeared in the Royal Academy last year, painted by I. Snowman, and entitled "The Leopard Skin." It represents a group of tiny children gazing with mingled alarm and curiosity at a leopard skin lying on the floor with the head directed towards the children.

### A NEW HAIR CURLER.

Lenton's patent hair curlers offer several advantages which especially recommend them to notice. They are extremely soft and pliable. The interior is composed of a number of strong but ductible metal wires covered by silk or cotton. They are circular in make, and there are no open prongs to tangle the hair. They are therefore very suitable for children's use. They may be had in any shade to match the hair, and are very inexpensive. There is also a "Lenton" hair fastener, which is practical and useful. The address of the firm is D. Lenton, Rockland Works, Coventry.

### A BABY'S OUTFIT.

I am glad to draw the attention of nurses to a layette, made of Dr. Lahmann's cotton wool, which I have been inspecting. For some time past a distinct desire for a more rational method of clothing infants has arisen, and nurses are frequently asked for advice on the subject. In summer, more especially, a difficulty arises, as woollen garments otherwise suitable, are so warm as to constitute a positive danger to children. Dr. Lahmann's soft, light, and elastic cotton material is a really ideal substitute. It is sufficiently warm and absorbent, and so soft and comfortable as to form an ideal clothing for a very young infant. The whole outfit is obtainable in the same material, and all the little garments are well finished and neat in appearance. If not as pretty as embroidered and belaced cambrics, the little day-dress for the baby which I have before me is so obviously formed for its comfort and health that no sensible mother would hesitate in her choice between the two. Every kind of garment for adults, male and female, are also procurable made of Dr. Lahmann's material. The address of the agency is 15 Fore Street, London, E.C.

### MESSRS. EGERTON BURNETT'S MATERIALS.

Nurses who are thinking of replenishing their wardrobes, or intended probationers buying uniform for the first time, should write to Messrs. Egerton Burnett, Ltd., Wellington, Somerset, for their patterns. I cannot too highly recommend the serges and galateas manufactured by this firm, which has been honoured by a large number of Royal and Imperial warrants of appointment, and which supplies many of the hospitals with materials for nurses' dresses and cloaks. For indoor uniform, washing drills and cottons are, of course, required, and these are made in all the styles usually worn, in plain blues, dark and light, pinks, browns, etc., as well as in stripes, checks and spots. The prices run from 7½d. per yard, and the materials are calculated to resist a great deal of hard wear. Grey cottons can also be had either plain or striped, as well as hollands, the latter ranging from 8½d. to 1s. 0½d. Linings for washing dresses are very moderate in price, being 6½d. or even 6d. per yard. A strong white linen is always required for nurses' aprons, and this is made in wide width, viz., 45 inches, from 1s. 6½d. per yard. Then for outdoor dresses, the serges manufactured by this firm are well-known for fast dye and durability; they range from 1s. 11½d. in navy blue, wide width, while I notice there is a charming grey alpaca at 2s. 4d. per yard; this quality is also made in dark blue, which is so generally worn, and which always looks neat and serviceable. A speciality is made of waterproof cloaking serges for out-door wear; these are specially dyed to prevent their turning a bad colour, and the price is from 3s. 3d. per yard. A tailor-made cloak can be supplied from 23s., to measure, the shape being, of course, indicated in ordering; the price I have mentioned is for a circular cloak in a plain yoke. In addition to materials for uniforms, Messrs. Egerton Burnett have just sent me a large selection of their new season's patterns for off-duty dresses. There are some charming cheviot tweeds and habit cloths, as well as light-coloured fancy materials, suitable for the still popular Russian blouse and skirt, one of the most effective being a costume cloth called the "Eashing," which is made in grey with a blue irregular stripe, green with a similar stripe of pink, and buff, relieved by a blue line. For a quiet serviceable dress, perhaps the grey tweeds and habit cloths can hardly be improved upon, and in this style there is abundance of variety to select from, while the mercerised linens, zephyrs, and voiles in black and delicate shades, offer a choice that is almost bewildering. I notice an inexpensive and useful canvas, 29 inches wide, from 8½d., and this I think would be very suitable to a warm climate, as well as for summer wear in this changeable British temperature. Then I must not omit to mention the dainty muslins and zephyrs, flowered, striped, spotted and plain, which make pretty blouses for spring and summer, nor the strong washing cottons for every-day use. I am also asked to draw attention to the ladies' tailoring department, which is said to be becoming increasingly popular: plain walking and cycling costumes, blouses, etc., are made to order, and patterns of materials, price-lists and self-measurement forms will be sent to any address. Canadian and South African nurses may like to know that there is a reduction of customs duty on British woollen goods, to which attention is drawn in the price-list before me. Collars and cuffs, gloves, and underclothing are among the details of out-fit supplied by this firm, as well as rugs, quilts, etc.

### Echoes from the Outside World.

### Movements of Royalty.

THE King and Queen held at Buckingham Palace on Friday night the first Court of the season, which had been postponed in consequence of the death of the Duke of Cambridge. The Royal circle was much smaller than usual owing to the absence of many members of the Royal Family from England. The King wore a military uniform. After the diplomatic presentations had been made, the *débutantes* passed before the King and Queen, who were supported by a brilliant retinue as well as by an exceptionally large body of the diplomatic corps. On Monday their Majesties left London for Ireland on the *Victoria and Albert*, arriving in Kingstown on Tuesday morning. They had a most enthusiastic reception, and the King, replying to addresses from various local bodies, expressed the pleasure which it gave to the Queen and himself to visit Ireland again, and the deep interest they felt in the efforts made to improve the condition of the working classes, and they hoped for beneficial results from recent legislation.

### The Prince and Princess of Wales at Vienna.

THE visit of the Prince and Princess of Wales to Vienna terminated on Saturday, when they left the Austrian capital for Stuttgart. The first day of the visit was devoted to a discharge of the social duties incumbent upon Royal visitors. On the second day they went, early in the morning, to the Spanish riding school, thence to the picture gallery in the Historical Museum of Art, and afterwards returned to the carriage and saddle-room of the Court stables, where historical coaches and past and present accoutrements were minutely inspected. They next drove out to the Freudenau racecourse, attended a gala dinner in the large Hall of Ceremonies, and afterwards paid a visit to the Opera. Then the Princess proceeded to a ball given in her honour by the Archduke Friedrich, and the Prince started by train for Neuberg. Here on the third day he shot several fine capercailzies. The Prince and Princess after luncheon at Schönbrunn witnessed an entertainment known as a "Pirutschade," in the course of which 15 carriages were driven through an expanse of spring flowers, chiefly violets and pansies. In the evening they dined at the British Embassy, and a reception followed. At Stuttgart, which the Prince and Princess reached on Saturday evening at nine o'clock, their Royal Highnesses were received at the railway station by the King and Queen of Würtemberg. On Monday evening the Prince formally invested the King of Würtemberg with the Order of the Garter in the throne room of the Royal Castle.

### The War in the Far East.

ANOTHER mine disaster occurred to the Russians at the close of last week. On Friday Admiral Alexeieff telegraphed to the Czar that while some mines were being laid by steam launches, one of them prematurely exploded and killed a lieutenant and 20 men. Admiral Alexeieff has also sent a detailed report of the circumstances attending the loss of the *Petropavlovsk* to the Czar, and a report of a skirmish in the vicinity of the Ya-lu in which the Russians lost 20 officers and men killed and wounded. It is stated that newspaper correspondents accredited to the Russian forces, who have started for Mukden, have bound themselves not to divulge news respecting the result of engagements with the enemy or the losses suffered by the Russians. Three Russian warships and two torpedo-boats entered Gen-san harbour on Monday, and in the afternoon the torpedo-boats sank a small Japanese merchant steamer. The squadron, which is reported to have come from Vladivostok, immediately afterwards left.

### The New Judge.

THE vacancy in the Chancery Division, owing to the death of Mr. Justice Byrne, has been filled by the appointment of Mr. Thomas Rolls Warrington, K.C. Little known to the general public, and quite unknown in the political world, the new judge, who was born in May, 1851, and created a Queen's Counsel in 1895, has won a high reputation in legal circles, both on account of his attainments as a lawyer and because of his unfailing courtesy to his opponents at the Bar. For some years he has been the leader in Mr. Justice Kekewich's Court, and he is a prominent Freemason.

### Great Fire in London.

A DISASTROUS fire broke out in the City of London just before midnight on Monday, and raged more or less fiercely until the middle of Tuesday morning. Before it could be extinguished the goods depôt of the London and North-Western Railway Company, which covered acres of ground between Aldgate and the Minories, with their contents, were almost entirely destroyed. It is calculated that upwards of 300 firemen, with 48 land steamers and long ladders, were engaged in the operations, and the casualties included injuries to two of the force. At three o'clock there was an unexpected development of the fire owing to the flames penetrating to the wine and whisky vaults in the sub-basement. From the lower floors blue and yellow masses of fire passed out in a remarkable manner, and the heat thrown out was extraordinary. The historic church of Holy Trinity, built in 1295, was at one time in danger.

### St. George's Day.

THE day devoted to the patron Saint of England happened this year to coincide with the celebrations in honour of Shakespeare and also with the date selected for the Cup Final Football Match between the Bolton Wanderers and Manchester City at the Crystal Palace. It was noticeable that a large proportion of the thousands of visitors from the North who came up to witness the match wore the national flower, and their example served to remind Londoners that they had forgotten their duty. Consequently, the rose was more conspicuous in the metropolis than on any previous St. George's Day. At Shoreditch there was a pretty revival of parish revels, and a procession composed of nearly 200 persons arrayed in the dresses of past ages, Maypole and Morris dancers, Knights in armour, famous historical characters, shepherds, and soldiers paraded the streets. At Stratford-on-Avon the old morris dancers were also revived with conspicuous success, and the great ceremony of the day was the laying of floral tributes on the poet's grave.

### The New Gallery.

NOW that Mr. Watts, as a retired Academician, is only allowed to send one picture to the Annual Exhibition at Burlington House, his wonderful exhibits at the New Gallery, of which this year there are five, are additionally interesting. Two of these five canvases treat of infant life. One represents a babe coming out of the sea, and is entitled "Whence? Whither?" the other, "A Fugue," is a spiral column of babies, mingled with flowers, being borne aloft. "Endymion," "Progress," and "Prometheus," are the other subjects depicted. Mr. Byam Shaw's "Last Days at Ludlow, 1483," shows Edward V. and his younger brother in a small room of the castle looking over the hills through barred windows, sadly pathetic little figures. Mr. Collier has a most successful full-length portrait of "Mrs. Anthony Hope Hawkins," and Mr. Shannon has never done better than in "Joan Ratcliffe," a little girl in a park in a white frock and a black cloak, carrying her doll. Lady Alma Tadema has an unpretentious charming picture of a Dutch interior, entitled "Sweet Industry," Mr. Harold Speed has given a soft and beautiful moonlight effect in "Chiaro di Luna," and Mr. J. W. North's "Little Rivers Rising in the West" is an admirable landscape.

### Notes and Queries.

**FOR REGULATIONS SEE PAGE 12.**

*Lecturer.*

(30) I shall be glad if you will tell me to whom I should apply for information as to the necessary steps to be taken in order to become a lecturer under the London County Council.—*A Trained Nurse.*

Apply to the London County Council, Spring Gardens, S.W.

*Milk Sterilisation.*

(31) I should be glad to know how milk could be sterilised at home. It is for a delicate baby in a household where want of means forbids any outlay.—*Nurse M.*

Put the milk to be sterilised into a bottle, place the bottle in a pan of cold water, let the level of the water in the pan be above the level of the milk in the bottle, and let the neck of the bottle be well out of the water and unstoppered. Bring the water to the boil, and keep it boiling for twenty minutes. Put the cork securely into the bottle just as the pan is about to be removed from the fire. If this method is properly carried out the milk will keep good for many days.

*Home.*

(32) Can you kindly tell me of a home where babies a few weeks old are received for a small payment weekly ?—*Dolores.*

Dr. Barnardo has a home for babies. Apply for information at Dr. Barnardo's Homes, 18–26 Stepney Causeway, E.

Can you tell me if there is a suitable working home or colony where the son of a gentleman, aged 21, could be received? He is strong and well but owing to a fall from the mast suffers from slight mental trouble ; though no doctor would give a certificate of lunacy. His parents are not well off but could contribute a small amount towards his support, and they are anxious that he should be physically employed. Am I right in thinking that there is a colony for epileptics in England, and if so, where ?—*Sister.*

Apply to the National Society for the Employment of Epileptics, The Colony, Chalfont St. Peters. Office, 12 Buckingham Street, Strand, W.C.

*Zenana Hospital.*

(33) Is there any hospital in connection with Zenana Missions in India ; and if so where can I obtain information ?—*E. D.*

Apply to the Zenana Bible and Medical Mission, 2 Adelphi Terrace, W.C.

*Nursing in Canada.*

(34) Will you tell me where I can obtain information as to nursing in Canada. I am anxious to go out when I shall have finished my training.—*E. R. S.*

Apply to the Lady Superintendent, Victorian Order of Nurses for Canada ; Headquarters, 578 Somerset Street, Ottawa, Ontario. See article in *Nursing Section* of Hospital for August 29th, 1903.

I should be glad if you will kindly tell me the best way of getting an appointment in Canada as I wish to settle there.—*Nurse M.*

See reply to *E. R. S.*

*Classes in Dublin.*

(35) Will you kindly tell me if there are classes held in Dublin to prepare pupils for the Apothecaries' Hall Examination ?—*A. A. D.*

Apply to the Secretary, Apothecaries' Hall of Ireland, 40 Mary Street, Dublin, for particulars as to examinations for the Irish certificate.

*Nursing Co-operative Associations.*

(36) Can you tell me if there is a nursing co-operative society at either Leicester or Birmingham ?—*E. M. F.*

The Nurses' Co-operation, 23 Francis Road, Edgbaston, Birmingham.

### Important Nursing Textbooks.

"The Nursing Profession : How and where to Train." 2s. net ; 2s. 4d. post free.

"A Handbook for Nurses." By Dr. J. K. Watson. 5s. net ; 5s. 4d. post free.

"Practical Guide to Surgical Bandaging and Dressings." By Wm. Johnson Smith, F.R.C.S. 2s. post free.

"The Nurses' Dictionary of Medical Terms and Nursing Treatment." By Honnor Morten. 2s. post free.

"Mental Nursing." By William Harding, M.D.Ed. 1s. post free.

"Art of Feeding the Invalid." (Popular Edition.) 1s. 6d. post free.

"On Preparation for Operation in Private Houses. By Stanhope Bishop, F.R.C.S. 6d. post free

"Modern Nursing in Private Practice." By Sir William Bennett. Price 7d. post free.

### For Reading to the Sick.

### A VISION OF SPRING.

I wandered lonely as a cloud
 That floats on high o'er vales and hills,
When all at once I saw a crowd,
 A host, of golden daffodils ;
Beside the lake, beneath the trees,
Fluttering and dancing in the breeze.

Continuous as the stars that shine
 And twinkle on the milky way,
They stretched in never-ending line
 Along the margin of a bay :
Ten thousand saw I at a glance,
Tossing their heads in sprightly dance.

The waves beside them danced, but they
 Outdid the sparkling waves in glee :—
A poet could not but be gay
 In such a jocund company.
I gazed, and gazed, but little thought
What wealth the show to me had brought

For oft, when on my couch I lie
 In vacant or in pensive mood,
They flash upon that inward eye
 Which is the bliss of solitude ;
And then my heart with pleasure fills,
And dances with the daffodils.

*Wordsworth.*

The following beautiful description of a daffodil meadow written by one who later became an invalid, and was herself also a keen observer of Nature, dwelling with loving insight on every detail of its varying moods may, we hope, be appreciated by the readers of this column by bringing before them a scene, which they may be unable to view otherwise than mentally.

"I sped on again, stray daffodils lighting the wayside, until I heard the voice of the stream, and reached the gate which leads to the lower meadows. There before me lay spring's pageant : green pennons waving, dainty maids curtseying, and a host of joyous yellow trumpeters proclaiming 'victory' to an awakened earth. They range in serried ranks right down to the river, so that a man must walk warily to reach the water's edge where they stand gazing at themselves in fair semblance like their most tragic progenitor, and, rising from the bright grass in their thousands, stretch away until they melt in a golden cloud at the far end of the misty mead. Through the field gate and across the road I see them starring the steep earth bank that leads to the upper copse, gleaming like pale flames against the dark tree-boles. There they have but frail tenure ; here in the meadows, they reign supreme. At the upper end of the field the river provides yet closer sanctuary for these children of the spring. Held in its embracing arms lies an island long and narrow, some 30 feet by 12, a veritable untrod Eldorado, glorious in gold from end to end, a fringe of reeds by the water's edge, and save for that—daffodils. . . . The air was keen and still as I walked back in the early evening, and a daffodil light was in the sky as if Heaven mirrored back earth's radiance."—*M. Fairless* in "*The Roadmender.*"

## Figures and Tables

**Figure f1:**
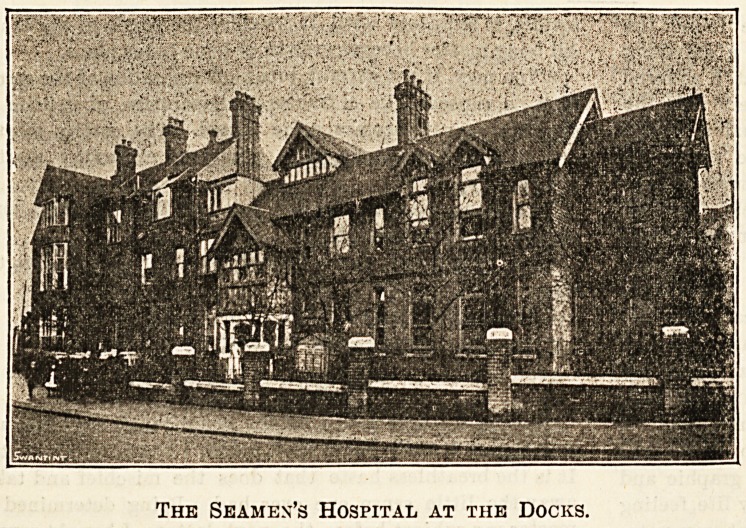


**Figure f2:**
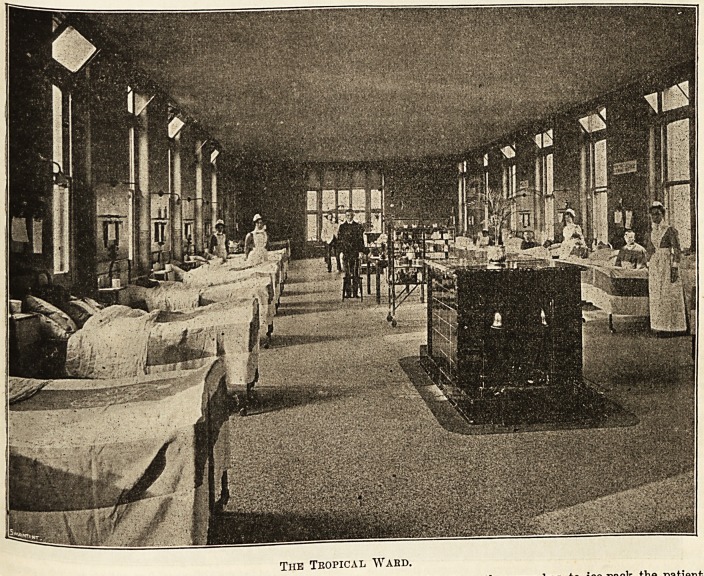


**Figure f3:**